# Self-care practices of people living with HIV and hypertension or diabetes comorbidities in low-and-middle income countries: A systematic review

**DOI:** 10.1371/journal.pone.0360076

**Published:** 2026-10-09

**Authors:** Andrew K. Tusubira, Andrew S. Ssemata, Anne R. Katahoire, Carl May, Christine K. Nalwadda, Moffat Nyirenda, Susannah Mayhew

**Affiliations:** 1 Department of Global Health and Development, Faculty of Public Health and Policy, London School of Hygiene and Tropical Medicine, London United Kingdom; 2 MRC/ UVRI and LSHTM Uganda Research Unit, Entebbe, Uganda; 3 Department of Community Health and Behavioral Sciences, Makerere University, College of Health Sciences, Kampala, Uganda; 4 Child Health and Development Centre, Makerere University, Kampala, Uganda; PLoS ONE, UNITED STATES OF AMERICA

## Abstract

**Background:**

People living with HIV in low-and middle-income countries (LMICs) face a growing burden of hypertension and diabetes comorbidities. Although self-care is essential, limited evidence exists on how patients manage these conditions concurrently within resource-constrained settings. We examined self-care practices among people living with HIV and hypertension or diabetes comorbidities in LMICs and identified treatment burdens related to these practices.

**Methods:**

We searched Medline, EMBASE, PsycINFO, Global Health, Scopus, Web of Science, CINAHL, WHO Global Index Medicus, African Journals Online and, Google Scholar for primary studies conducted in LMICs and published between January 2013 and March 2026, regardless of study design and data collection method. Two reviewers independently screened, extracted data, and assessed study quality, resolving discrepancies through discussions. We used thematic synthesis to analyze self-care practices, and mapped findings onto the Normalization Process Theory to identify associated treatment burdens. Confidence in the evidence was assessed using the GRADE-CERQual approach.

**Results:**

Thirteen qualitative studies from six African countries, comprising 458 participants, were included. Seven studies were rated high quality while six were moderate. Five themes emerged with moderate-to-high confidence in evidence: 1) Sense-making of illnesses and medications; 2) Divergent treatment approaches between HIV and hypertension or diabetes treatment; 3) Adoption of health-promoting behaviors, including lifestyle changes; 4) Coping with psychosocial distress, and 5) Strategies to navigate healthcare insecurities. Related treatment burdens were linked to prescribed healthcare tasks, disparities between HIV and hypertension/diabetes care, and patient-initiated efforts to compensate for gaps in hypertension/diabetes care.

**Conclusion:**

Self-care practices were diverse and shaped by individuals’ interpretations of illnesses, structural inequities, and the cumulative demands of multimorbidity. Strengthening consistent education on illness and medications, psychosocial support, and lifestyle guidance can improve equitable integrated care and support effective self-care. Future research should quantify self-care workload and treatment burden and evaluate integrated care models that reduce patient workload.

**Systematic review registration:**

PROSPERO: CRD42023463738.

## Introduction

Non-communicable diseases (NCDs), particularly hypertension (HTN) and diabetes mellitus (DM), are increasingly prevalent among people living with HIV (PLHIV) in low- and middle-income countries (LMICs) [1,2]. Projections suggest that up to 84% of PLHIV will develop at least one NCD by 2030 [3]. Globally, about 24% of PLHIV live with HTN and 9% with DM [4,5], with nearly 59% of those with HTN residing in sub‑Saharan Africa and HIV/DM comorbidity reaching up to 14.9% in some countries [1,4,6,7]. This growing multimorbidity is attributed to increased life expectancy from effective antiretroviral therapy (ART), ART-related metabolic effects, HIV-associated inflammatory and vascular changes, and lifestyle risk factors [8–10]. Coexisting HTN or DM complicates HIV care through polypharmacy, overlapping symptoms, and frequent hospitalizations, thereby reducing quality of life, heightening mortality and increasing economic strain on patients, caregivers, and health systems [11]. Consequently, global and national strategies now emphasize integrated, patient-centered care models to address multimorbidity [12].

Self-care is a core component of chronic disease management, enacted through informed decision-making, daily healthy behaviors and adjustments [13,14]. Self-care is broadly defined as the ability to care for oneself to achieve, maintain, or promote optimal health and well-being [13]. However, in LMICs, socio-cultural norms, financial constraints, and health system limitations influence how chronic conditions are managed [15,16]. In the context of multimorbidity, such as HIV and HTN or DM, self-care extends beyond adherence to a single treatment regimen to encompass continuous decision-making and a wider range of patient-driven activities embedded in daily life [17]. Moreover, health systems are increasingly shifting routine care tasks to these patients, placing substantial care burdens on them [18]. It is therefore critical to understand how they navigate these intersecting demands to design feasible, patient-centered interventions that fit patients’ real-life contexts.

Efforts to integrate HTN and DM services into HIV platforms have expanded across LMICs, with evidence of benefits such as staff capacitation, improved screening, detection and continuity of NCD care [19,20]. However, HIV/NCD integration remains uneven and constrained by shortages of NCD medicines, infrastructure, clinical guidance, and human resources [19,21]. Consequently, more than half of PLHIV with HTN or DM have uncontrolled blood pressure or glucose despite good HIV outcomes among the same patients [22,23], highlighting persistent gaps in both service delivery and patient self-management.

Effective self-care improves adherence and clinical outcomes while reducing health system burden, but it requires understanding patients’ lived experiences, challenges, and coping strategies [24,25]. For people living with multimorbidity, treatment burden further undermines their quality of life and overall health outcomes [18]. However, little is known about how PLHIV with HTN or DM concurrently navigate self-care demands within resource-constrained settings, and how these demands accumulate into treatment burden. This gap limits the design of feasible, patient-centered interventions and poorly aligned self-care demands can increase treatment burden, negatively affecting outcomes for both conditions. We therefore aimed to examine self-care practices among PLHIV with HTN or DM in LMICs, with a secondary aim of identifying the treatment burdens associated with these practices. The Normalization Process Theory (NPT) guided the identification of the treatment burdens [26,27]. NPT was selected because it provides a structured, empirically validated framework for understanding the work patients undertake to enact healthcare practices and has been applied to multimorbidity research in LMICs [28,29]. Unlike the Burden of Treatment Theory [30], which focuses primarily on capacity-demand imbalances, NPT enables systematic mapping of the specific tasks and processes through which treatment burdens arise and are sustained in everyday life [26,27]. Findings are intended to inform clinicians to improve patient self-management for multimorbidity, guide program implementers in strengthening self-care in HIV/NCD integrated care and identify research gaps to support the development of sustainable, patient-centered self-care strategies.

## Methods

### Design

A qualitative systematic review using thematic synthesis conducted in accordance with the Preferred Reporting Items for Systematic Review and Meta-Analysis (PRISMA) 2020 reporting guidelines and the Enhancing Transparency in Reporting the Synthesis of Qualitative Research (ENTREQ) statement [31]. The complete PRISMA and ENTREQ checklists are provided in S1 Table and S2 Table, respectively. The review was conducted between September 2023 and March 2026 and was registered with the International Prospective Register of Systematic Reviews (PROSPERO: CRD42023463738). Although the original protocol anticipated inclusion of qualitative, quantitative, and mixed-methods studies, only qualitative studies met the final inclusion criteria. Consequently, the review design and synthesis focused exclusively on qualitative evidence.

Our primary review question focused on exploring self-care practices among individuals living with HIV and comorbid HTN or DM in LMICs. During synthesis, the demands associated with managing multiple illnesses consistently emerged across studies. In line with qualitative methodology [32], we iteratively expanded the analysis to identify these demands as treatment burden by mapping our thematic analysis findings onto the NPT [26,27]. NPT is a structured framework for understanding treatment burden and has been applied to multimorbidity research in LMICs [28,29]. Treatment burden was conceptualized as the healthcare workload or tasks that patients do to manage their conditions, consistent with established definitions [18,33]. Mapping findings onto this framework enabled systematic interpretation of how self-care practices translate into everyday burdens across patients’ lives.

### Eligibility criteria

#### Population.

Eligible studies included adults (≥18 years) living with HIV and HTN or DM or both comorbidities, irrespective of whether HTN or DM onset preceded or followed HIV infection. We focused on PLHIV because they represent a growing population experiencing chronic multimorbidity and they have established care platforms which can influence self-care. Adults were included because self-care responsibilities and treatment decision-making differ substantially from those of children and adolescents. We did not restrict inclusion by duration of diagnosis or treatment status (e.g., receiving treatment or not), as these characteristics were inconsistently reported and were not central to the review objective.

#### Phenomenon of interest/outcome.

We included studies reporting self-care practices or behaviors undertaken by individuals to manage HIV and comorbid HTN and/or DM. The primary outcome of this review was self-care practices for managing HIV and HTN or DM multimorbidity in LMICs. Self-care practices were defined as health-related actions or measures undertaken by individuals to manage illness, maintain health, or restore well-being, encompassing physical, psychological, spiritual and social strategies [34]. This review did not include studies reporting clinical or disease outcomes among PLHIV and HTN or DM comorbidities.

#### Type of studies/ study design.

We included primary studies employing qualitative, quantitative or mixed methods designs. Eligible quantitative studies comprised observational studies (cross-sectional, cohort and case-control studies), interventional studies and surveys. Qualitative studies were eligible regardless of design or data collection method. We excluded secondary research papers (including systematic reviews and protocols), policy analyses, editorials, commentaries, abstracts, conceptual papers, and studies involving non-human participants.

#### Context.

Only studies conducted in LMICs were included. We focused on LMICs because distinct health system constraints, resource limitations, and the evolving integration of HIV and NCD care shape self-care practices in these settings. Therefore, patient experiences and challenges in LMICs differ markedly from those in high-income counties, influencing how self-care is understood and practiced. A summary of inclusion and exclusion criteria is provided in Table 1.

**Table 1:** Review inclusion and exclusion criteria.

Inclusion criteria	Exclusion criteria
**Outcome/ Phenomenon of interest**	
Studies focused on self-care practices among PLHIV and HTN or DM comorbidities.	Studies reporting on disease outcomes for HIV, HTN or DM. Studies among PLHIV and HTN or DM with outcomes not related to self-care.
**Population**	
Studies that included adult participants (18 years and above) living with HIV and HTN, or DM, or HTN and DM comorbidities.	Studies that focus on the population of PLHIV only. Studies that focus on the population of people with HTN only, or that focus on the population of people with DM only.
**Type of studies**	
Qualitative studies, quantitative studies and mixed-methods studies on humans.	Studies reporting qualitative or quantitative data on nonhumans.
Observational studies (cross-sectional, cohort and case-control studies); interventional studies; and surveys.	Systematic reviews, protocols, policy analyses; case reports; abstracts; conceptual frameworks and/or theories; commentaries and editorials.
**Context**	
Studies conducted in the LMICs	Studies conducted in developed countries

HTN – hypertension; DM – diabetes mellitus.

#### Data sources.

We searched the following databases: Ovid Medline, Ovid EMBASE, Ovid Global Health, Ovid PsycINFO, Web of Science (including Social Science Citation Index), Scopus, CINAHL via EBSCO, WHO Global Index Medicus, Google Scholar and African Journals Online (AJOL). The AJOL was included to capture African research not indexed in major databases.

#### Search strategy.

Search terms were developed in consultation with an information specialist at the London School of Hygiene and Tropical Medicine (LSHTM). The search strategy combined three key concepts and their synonyms: “self-care”, “human immunodeficiency virus,” and “hypertension” or “diabetes”, using Boolean operators. We modified search terms as required by each database and used both keywords and medical subject headings (MeSH). Some of the MeSH terms were: “Self-Care”, “Self-manage”, “self-monitor”, “self-medicate”, “adherence”, “PLWHIV”, “PLHIV”, “HIV”, “HIVAIDS”, “HIV Infection”, “acquired immune deficiency syndrome” “SIDA” “Hypertension”, “Blood Pressure”, “Diabetes”, “Diabetes Mellitus”, “high blood sugar”, “hyperglycemia”. Reference lists of included studies were also searched for possible papers. Given AJOL’s limited search and export functionality, a manual search was done and retrieved titles and abstracts. The literature search covered studies published between January 2013 and March 2026 in LMICs. The review involved a two-stage search process. An initial data search was conducted in December 2023. To ensure that the review incorporated the most recent evidence prior to submission, we conducted a final update of the literature search in March 2026 by re-running the same search strategies across all databases. Newly identified records were screened using the same eligibility criteria and incorporated into the final synthesis. The period from 2013 was chosen because it captures both the expansion of self-care concepts in literature and a period during which HIV/NCD integration has evolved [35,36]. There were no language restrictions to maximize the scope of relevant articles from LMICs. Full search strategies, including MeSH terms, are provided in S3 Table.

#### Study screening and selection.

Following the search, all citations were imported into EndNote X20 software (Clarivate Analytics) [37], and duplicates removed. Screening was managed using Rayyan software [38]. Two reviewers (AKT and ASS) independently screened titles, abstracts, and full texts. We resolved discrepancies through discussion and by involving a third reviewer (SM).

#### Data extraction.

Two reviewers (AKT and ASS) independently extracted the data using an extraction form guided by Cochrane [39,40], with discrepancies resolved through discussions with another reviewer (SM). Extracted data included authors and year of publication, country, study aim, study design, participant characteristics (including age and sex), participant NCD comorbidity, sample size, data collection and analysis methods, and key findings. We also extracted texts, quotations and relevant data on self-care practices.

#### Inter-rater reliability.

Study selection and data extraction were performed independently by two reviewers. Inter-rater reliability was assessed using Cohen’s kappa. For full-text study selection, (36 articles assessed; 13 included) the kappa value was 0.84, and for data extraction across double-extracted items, kappa value was 0.80, indicating substantial agreement between reviewers.

#### Quality assessment.

Since only qualitative studies were included, we used the critical appraisal skills program (CASP) appraisal checklist for qualitative studies to assess the quality of the studies [41]. CASP checklist for qualitative research is a widely used critical assessment tool for evaluating the quality and relevance of qualitative research by assessing their methodological rigor (including assessing key aspects like recruitment, data collection, and analysis) and ensuring that ethical standards were met. Two reviewers independently assessed each article and assigned an overall quality ranking of “low”, “medium”, or “high” quality. We followed the score and classification system suggested by Butler *et al* [42]. Hence, every “Yes” scored 1 point, every ‘Can’t tell or Not sure’ scored 0.5 points and every ‘No’ scored 0. Articles were then classified into three categories following their total score: 1) high-quality paper (scored between 9.5 and 10), moderate-quality paper (scored between 7.5 and 9), low-quality paper (scored less than 7.5) and those under 6 points were to be excluded. Consistent with qualitative evidence synthesis methodology, no studies were excluded based on quality assessment scores, as excluding studies solely on methodological quality grounds may remove contextually rich evidence and introduce selection bias [43]. Instead, quality appraisal was used to assess methodological rigor and transparency and to inform the interpretation of the review findings through the Grades of Recommendation, Assessment, Development and Evaluation – Confidence in Evidence from Reviews of Qualitative Research (GRADE-CERQual) approach [44,45]. Notably, after the appraisal, all included studies were rated as either high or moderate quality, and no low-quality studies were identified.

#### Data analysis.

Since only qualitative studies were included, we conducted thematic synthesis following Thomas and Harden’s three stages: 1) line-by-line coding; 2) development of descriptive themes; and 3) generation of analytical themes [43]. The coding framework was developed iteratively. Initial line-by-line coding was conducted independently by two reviewers (AKT and ASS) on a subset of included studies to develop and pilot the preliminary coding framework. The two reviewers compared their coding, discussed differences in interpretation, and refined the coding framework through consensus. This piloting phase allowed reviewers to test and calibrate the coding approach, identify areas of ambiguity, and establish a shared understanding of the coding categories before proceeding to the full data set.

The revised framework was then applied to all included studies and further refined iteratively as new codes and patterns concepts emerged from the data. Regular discussions between reviewers were held throughout the synthesis process to ensure consistency, challenge assumptions, and maintain reflexivity in interpreting the findings. Discrepancies in coding or thematic interpretation were resolved through discussion and consensus, with consultation from a third reviewer (SM) when agreement could not be reached. To address reflexivity and minimize reviewer bias, the team (AT, AS, SM and AK) maintained ongoing critical dialogue throughout the synthesis process, reflecting on how prior assumptions and disciplinary perspectives might influence interpretation of the data. This reflexive approach was embedded throughout the analysis rather than a single discrete step.

This inductive approach enabled reviewers to move beyond descriptive findings to generate interpretative and analytical insights. Descriptive themes of self-care practices were then generated to summarize the meaning of the initial codes and code groups. These descriptive themes were further interpreted and organized into analytical sub-themes and overarching themes, providing a deeper understanding of the data.

Although the review protocol planned to include both qualitative and quantitative studies, our search yielded only qualitative evidence. Consequently, the synthesis focused exclusively on qualitative findings using thematic synthesis, rather than narrative synthesis, or meta-analysis. All eligible qualitative findings were included to ensure comprehensive and unbiased synthesis.

#### Contributions of studies to the final thematic findings.

We assessed each study’s relative contribution to the final synthesized results: (a process proposed by Thomas and Harden [43]). We used the subthemes of our thematic analysis to determine each study’s contribution. These sub-themes represented granular analytical insights, allowing us to evaluate how each study informed the overall synthesis. Since our review had 17 sub-themes, a score of one meant that the study provided information to one sub-theme, while a score of 17 meant that that study contributed to all the sub-themes of this review. (This approach has been used in prior review studies to score the contribution of individual papers to review findings [46,47]).

#### Confidence in the findings and identifying the treatment burden.

We used the GRADE-CERQual approach to assess the level of confidence in each review finding under its respective theme [44,45]. We first created a summary of review findings, then evaluated each review finding across the four GRADE-CERQual components: 1) methodological limitations, 2) coherence, 3) adequacy of data, and 4) relevance [44,45]. For each component, we judged the level of concern (in terms of no/very minor, minor, moderate, or serious concern), and used these judgements to assign an overall confidence rating (high, moderate, low or very low), starting with high confidence and downgrading as concerns increased [44,45]. Confidence judgments were reached through discussion and consensus between two reviewers (AKT and ASS).

To identify treatment burdens, findings with high or moderate confidence were onto the NPT four domains: coherence, cognitive participation, collective action, and reflexive monitoring [26,27]. Finding that did not align with these domains were categorized as “unallocated” following Chikumbu et al. [28]. We identified specific treatment burdens under each domain by capturing demands and tasks described to take patients’ time and efforts in each review finding.

## Results

Our search yielded a total of 8,572 records. After removing duplicates, 3,959 titles and abstracts were screened, and 36 articles were retrieved and underwent full text review. Twenty-three were excluded, leaving 13 studies for data extraction and analysis, as shown in Fig 1. Reasons for full-text exclusions are provided in the S4 Table.

**Fig 1:**
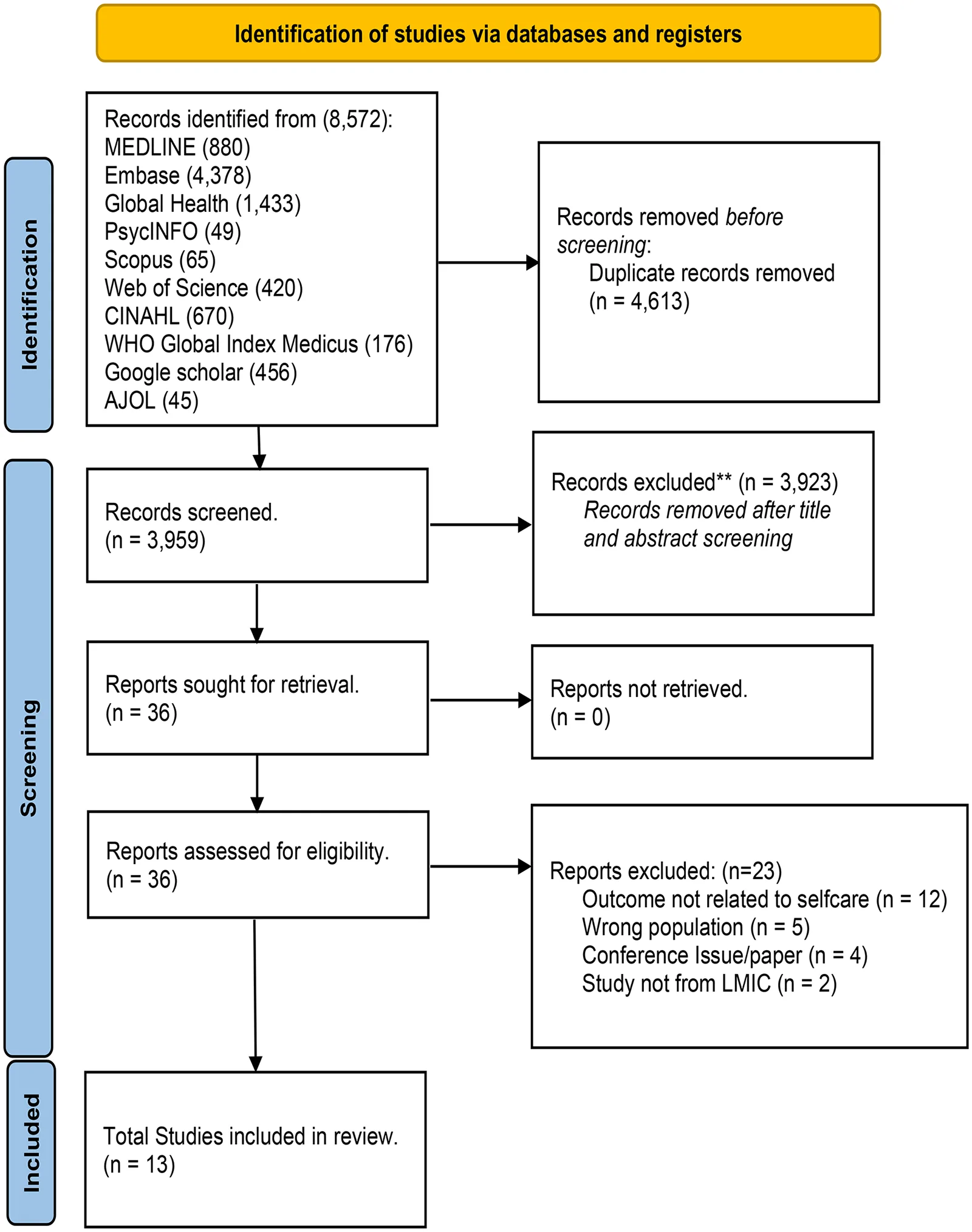
The PRISMA chart showing exclusion and inclusion of studies.

### Setting and characteristics of included studies

The 13 included studies were published between 2017 [48] and 2025 [49] and were conducted in six African countries: South Africa (four studies [50–53]), Uganda (two studies [54,55]), Malawi (two studies [56,57]), Kenya (two studies [48,58] Tanzania (two studies [59,60]), and Côte d’Ivoire (one study [49]). All were qualitative in design. Data collection methods included in-depth interviews (IDIs) or semi-structured interviews in nine studies employed [49–51,53,55–57,59,60]; narrative interviews in two studies [52,58]; focus group discussions in one study (FGD) [48]; and a combination of both IDIs and FGDs in one study [54]. The sample sizes ranged from six [55,58] to 100 participants [58], with ages spanning 18–35 years [58] to 72 years [56]. One study included only women participants [55], while the remaining had both sexes. Four studies focused on PLHIV with HTN [51,56,57,59], three studies on PLHIV with DM [49,52,53], and six on PLHIV with HTN and DM multimorbidity [48,50,54,55,58,60]. Detailed study characteristics are presented in Table 2.

**Table 2:** Summary of included studies (N = 13).

Author (Year), and country	Study Aim	Study design	Participant Characteristics (age, sex, and other social demographics)	Participant NCD comorbidity	Sample size	Data collection method	Data analysis method	Themes or key Findings
Hing et al., 2019, [56] Malawi	To identify and explore factors influencing adherence among individuals with HIV and hypertension in Malawi.	Qualitative study	Participants median age 53; Adult s with HIV and HTN; prescribed ART at least 1 year; prescribed HTN medication at least 1 year	HTN	75	Semi-structured interviews,	inductive and deductive analysis approaches	- Perceived susceptibility to HIV and hypertension - Perceived severity of HIV and hypertension - Perceived benefits of controlling HIV and hypertension - Perceived barriers to controlling HIV and hypertension - Perceived self-efficacy in controlling HIV and hypertension - Cues to action for controlling HIV and hypertension
Bosire et al., 2018 [58], Kenya	To investigate how biological sub-citizenship fundamentally shifts how people navigate the health care system and manage other diseases, with a special focus on diabetes.	Qualitative, life history narrative	Adults; 50% Male, 50% female; primary or less 51%, secondary or more 49%; employed 75%	HTN, DM	Focus on three vignettes, although 100 interviewed	Narrative interviews	inductive approach	- Description of “Biological sub-citizenship”, where HIV care services are consistent and comprehensive, with free medical care, while for DM services, patients must pay out-of-pocket for care. - The systematic exclusion from international donor funding creates, internal sociopolitical hierarchies, or a form of biological sub-citizenship, that may or may not have existed prior to the arrival of such funding.
Bosire, 2021 [52], South Africa	To explore patients’ experiences of accessing health care for comorbid HIV/AIDS and diabetes in Soweto, and how they self-managed their concurrent chronic illnesses at home.	Qualitative, ethnographic study	Patients above 30 years; receiving treatment for both HIV and DM. More than half of the participants were unemployed	DM	15	Narrative interviews and Observations	inductive approach	- Patients’ experiences of accessing chronic care in Soweto and - Patients’ experiences of self-management at home.
Akugizibwe et al., 2023, [54] Uganda	To illustrate the various dimensions of stigma and dynamics associated with integrated care of HIV, diabetes and hypertension in Ugandan clinics	qualitative, observational design.	30 patients (12 men and 18 women) (3 had HIV/HTN, 6 DM/ HTN, 2 HIV and DM, and 1 all three conditions). Other participants were: 10 healthcare providers; 5 policy makers; 10 community members; 5 community leaders; 1 representative from international NGO; and 2 clinical researchers	HTN, DM, HTN/DM	63	In-depth Interviews, Focus group	Thematic analysis	-Diverse multi-stakeholder perceptions around HIV related stigma and benefits of integration in chronic diseases management.
Manavalan et al., 2020 [59], Tanzania	Exploring the perspectives and experiences of patients with both HIV and hypertension in northern Tanzania to better understand barriers to hypertension management among PLHIV	Qualitative study	Participants with HIV and HTN, median age: 54. Eleven of the 13 were female. Nine attained primary education and 3 no education; 7 were widowed, 1 single and 3 divorced. Only one was a current user of HTN medicines.	HTN	13	In-depth interviews	Thematic analysis	*Themes related to patient perspectives:* -Limited knowledge of hypertension. -Perceived overlap and comparisons between hypertension and HIV. *Themes related to patient experiences:* -Delays in hypertension diagnosis and linkage to care. -Challenges with provider communication and counselling. -Reluctance towards antihypertensive medication. -Lack of integration for hypertension and HIV care. -Additional structural barriers to hypertension care.
Moucheraud et al., 2022 [57], Malawi	To understand HIV-positive Malawian adults’ experiences with hypertension management	Qualitative study	Most participants were female (n = 21); median age was 58; All men, versus only one-third of women, were married.	HTN	30	qualitative interviews	Thematic analysis	*- Themes about beliefs include* awareness of hypertension, attitudes toward the benefits of treatment, and opinions about the importance of medication adherence. *- Themes about behaviours include* social support for adherence, reasons for skipping or stopping medication, experience with medication side effects, use of complementary treatments, accommodating dietary changes, making changes to social relationships, and experiences with altered employment due to hypertension.
Matima et al., 2018 [53], South Africa	To the examination of individual’s experiences of HIV/T2D multimorbidity in the South African context	qualitative study	10 were patient participants and 6 healthcare workers. Patient-participants’ demographics: Eight had attained secondary school education, 50% were unemployed and 70% lived in informal housing − 7 were on insulin injections with 4 of them taking extra oral pills. The remaining 3 were taking pills only. -mean age = 46.9 years (Standard deviation 8.8). − 8 had secondary school level education, one primary level and one with tertiary qualification. − 7 of the 10 lived in informal housing. −6 had self-reported HTN	DM	16	In-depth interviews, Semi-structured Interviews	Thematic analysis	Two themes: Theme 1: Perceived patient workload (Clinic-related workload, Self-management workload). Theme 2: Perceived patient capacity
Van Pinxteren et al., 2023 [50], South Africa	To identify, characterize, and understand the workload and capacity associated with self-management amongst people with multimorbidity living in precarious circumstances in urban and rural South Africa.	Comparative qualitative study	22 HTN, 4HTN/DM; 2 DM; 1 Epilepsy; and 1 heart condition. Nine men and 21 women; mean age was 56 years in Gugulethu and 50 years in Bulungula.	HTN, DM, HTN/DM	30	Semi-structured interviews	Both inductive and deductive analysis approaches	- Treatment burden and the experiences of participants in Gugulethu and Bulungula, under the following domains: (1) control conditions and enacting control, (2) structural factors, (3) patient capacity, (4) quality of life, (5) impact of COVID-19
Temu et al., 2017 [48], Kenya	To explore lay beliefs about hypertension among HIV-infected adults to inform the development of culture sensitive hypertension prevention and control program.	Descriptive qualitative study	Of the 53 participants, 31 were women, 29 aged between 36&50 years, 12 between 18&35 years. 24 had at least postsecondary education, 18 primary level and 10 no education. Eleven of the 53 had HTN and 9 of the 11 had been prescribed HTN medicines: only 2 taking the meds. 3 of the 53 had DM.	HTN, DM	53	Focus group	Both inductive and deductive analysis approaches	Patient perspectives on hypertension: causes, complications and treatment. -Perceived causes. -Perceived complications. -Perceived treatment.
Tokwe and Naidoo, 2020 [51], South Africa	To explore and describe the lived experiences of people living with HIV (PLHIV) and hypertension in the Eastern Cape, South Africa.	Qualitative, using phenomenological data analysis	PLHIV and HTN for at least a year and 40year and above	HTN	9	Semi-structured interviews	Phenomenology/ inductive approach	*Four themes:* - Overcoming illness-related stigma - Sources of support - Self-love: taking ownership of diseases, and - Creating transforming behaviors and self-care strategies.
Van Hout et al., 2023 [55], Uganda		Qualitative, using phenomenological approach	Women aged between 42 and 55 years; 5 of the 6 diagnosed with HIV in less than 10 years. 2 with HIN, 2 with DM and 2 with HTN&DM comorbidities.	HTN, DM HTN/DM	6	In-depth interviews	Thematic analysis	Five themes: - Lay health knowledge and alternative medicine, - Community stigma, - Experiences of integrated care, - Navigating personal challenges, - Health service constraints.
Ottaru et al., 2024 [60], Tanzania	To describe lived experiences, challenges, and coping strategies for accessing care for hypertension and/or diabetes in HIV care and treatment clinics and other healthcare settings.	Qualitative study using a phenomenological approach	Participant age ranged from 34 to 75 years (median age was 56 years). 16 had HTN, 10 had DM and 7 had both.	HTN DM	33	Semi-structured interview	Thematic content analysis	Themes were in line with access to care for HTN/DM and coping strategies among the patients: Availability. Accommodation Affordability Acceptability Accessibility, and Copying strategies
Koffi et al., 2025. [49] Côte d’Ivoire	To examine the perceptions and management of HIV and diabetes mellitus among PLHIV and healthcare workers in Côte D’Ivoire.	Qualitative	Participant age ranged from 50 to 62 years. Nine were female and 6 males.	DM	15	Semi-structured interviews	thematic analysis	Three key themes: - Diabetes is perceived as a serious disease and a considerable burden for PLHIV, - Obesity is not perceived as a health problem, - Barriers to physical activity and healthy diet to manage diabetes and obesity

### Quality assessment results

For the quality of the included studies, most studies (seven of the thirteen) were of high quality [48,50,52,53,56,57,59], while the remaining studies [49,51,54,55,58,60] were of moderate quality. Concerning the relative contribution of each study, by and large the higher the quality of the paper, the more it was able to contribute to the review, with a few exceptions, as shown in Table 3.

**Table 3:** Quality assessment and relative contributions.

No.	Study ID	Q1	Q2	Q3	Q4	Q5	Q6	Q7	Q8	Q9	Q10	Total	Classi-fication of quality	Relative contri- bution
1	Van Pinxteren et al., 2023 [50]	Y	Y	Y	Y	Y	CT	Y	Y	Y	Y	9.5	H	12
2	Tokwe and Naidoo, 2020 [51],	Y	Y	Y	Y	Y	N	Y	CT	Y	Y	8.5	M	7
3	Moucheraud et al., 2022 [57],	Y	Y	Y	Y	Y	Y	Y	Y	Y	Y	10	H	10
4	Matima et al., 2018 [53],	Y	Y	Y	Y	Y	Y	Y	Y	Y	Y	10	H	9
5	Hing et al., 2019 [56],	Y	Y	Y	Y	Y	CT	Y	Y	Y	Y	9.5	H	6
6	Bosire et al., 2018 [58],	Y	Y	Y	CT	Y	CT	CT	Y	Y	Y	8.5	M	6
7	Temu et al., 2017 [48],	Y	Y	Y	Y	Y	Y	Y	Y	Y	Y	10	H	6
8	Manavalan et al., 2020 [59]	Y	Y	Y	CT	Y	Y	Y	Y	Y	Y	9.5	H	14
9	Akugizibwe et al., 2023 [54],	Y	Y	Y	CT	CT	N	Y	Y	Y	Y	8	M	4
10	Van Hout et al., 2023 [55],	Y	Y	Y	CT	CT	N	Y	CT	Y	Y	7.5	M	3
11	Bosire, 2021 [52],	Y	Y	Y	Y	Y	Y	Y	Y	Y	Y	10	H	9
12	Ottaru et al., 2024 [53,60]	Y	Y	Y	Y	Y	N	Y	Y	Y	Y	9	M	9
13	Koffi et al., 2025 [49]	Y	Y	Y	CT	Y	N	Y	CT	Y	Y	8	M	6
Total on each question		13	13	13	10.5	12	6.5	12.5	11.5	13	13			

Y = Yes; CT = Can’t tell; N = no; H = High; M = Medium; L = Low.

CASP questionnaire for qualitative studies: Q1. Was there a clear statement of the aims of the research? Q2. Is a qualitative methodology appropriate? Q3. Was the research design appropriate to address the aims of the research? Q4. Was the recruitment strategy appropriate to the aims of the research? Q5. Was the data collected in a way that addressed the research issue? Q6. Has the relationship between researcher and participants been adequately considered? Q7. Have ethical issues been taken into consideration? Q8. Was the data analysis sufficiently rigorous? Q9. Is there a clear statement of findings? Q10. How valuable is the research?

### Results from the thematic analysis

Five key themes emerged from the thematic synthesis: 1) Sense-making of illnesses and medications through grading and prioritization; 2) Divergent treatment approaches for HIV and NCD comorbidities amid polypharmacy; 3) Adoption of health-promoting behaviors; 4) Coping with psychosocial distress; and 5) Strategies to navigate healthcare insecurities. Themes are presented to reflect progression from pharmacological self-care practices (themes 1 & 2) to non-pharmacological and system-level strategies (themes 3, 4 & 5). Details of the themes and corresponding quotations are in S5 Table.

### Theme one: Sense-making of illnesses and medications by grading and prioritizing them

Sense-making of illnesses and medications emerged as a foundational self-care practice through which patients interpreted disease severity, evaluated treatment effectiveness, and prioritized care activities, influencing subsequent medication use, lifestyle adjustments, and coping strategies.

#### Grading and classifying the severity of illnesses.

Patients commonly assessed and ranked their condition based on perceived severity. Across several studies, HIV was described as a less severe, and more manageable illness compared with HTN or DM, which were viewed as unpredictable and potentially fatal [48–51,56,58]. This perception shaped treatment priorities and adherence behaviors. In contrast, HTN and DM were associated with sudden death, reinforcing fear and urgency in their management [56,58–60]:

*“The dangerous disease nowadays is blood pressure, because even sitting here, … I can die. While HIV, you stay longer “* (Female, 70 years old; HTN [56]).

#### Greater confidence in antiretroviral therapy than in NCD medications.

Patients consistently expressed strong trust in ART, describing ART as highly effective, reliable, and lifesaving [56,57,59]. This confidence fostered strong adherence to ART and optimism towards HIV care [50,52,56,57,59]. In contrast, medications for HTN and DM were seen as less effective, prompting doubts in efficacy and, use of herbal remedies to manage their NCD comorbidities [56,59,60]:

*“I went to the herbal clinic in search of an alternative, hoping to get treated. Because all these medications (hypertension and diabetes medication) are just for pain relief and not curative. The herbal clinic has medications that could cure diabetes completely”* (Male, 60 years; HTN).

#### Prioritizing care for HTN and DM comorbidities.

Grading illnesses influenced how patients allocated their time, emotional effort, and limited financial resources towards care [48,51,56,58,59]. Because HIV care routines were well-established, participants prioritized HTN and DM management due to their perceived immediacy and danger [51,58,59]. Some patients turned to dietary changes and traditional remedies, but they still indicated frustration due to poor health outcomes for the NCDs [50,51]:

*“With pressure, it does not go down …, I try to eat things I am told to eat but it is always high.”* (Female, 48 years old; HTN [51]).

### Theme two: Divergent treatment approaches for HIV and NCD comorbidities amidst managing polypharmacy

Building on patients’ sense-making of illnesses and medications, participants described distinct treatment approaches for HIV compared with HTN and DM. While ART was consistently emphasized as the central treatment for HIV, multiple approaches, including alternative therapies, were used for the comorbidities.

#### Consistent ART adherence versus intentional or selective non-adherence to NCD medication.

Patients demonstrated strong adherence to ART, which was widely perceived as lifesaving and easy to manage through routine pill-taking [49,50,52,56,57,59]. The free availability and reliable access to ART further reinforced consistent use [50,52,56,58]. In contrast, adherence to HTN and DM medications was consistently lower. Although none of the studies quantified this discrepancy, the pattern of non-adherence to NCD medications was observed, even where NCD medicines were accessible [48,52,57–59]. Non-adherence to NCD medicines was linked to the absence of symptoms, inconsistent advice from health workers, medicine stock-outs and side effects [48,52,57–60]:

*“I… do not use insulin so much because it affects my legs. They get [so] swollen that I can’t walk. So, I use it some days and skips other days”* (Female, 50 years; HTN&DM [58]).

A few patients with reliable access expressed strong commitment to adherence, often motivated by personal or family health histories [48,56]:

*“My father died of hypertension, and uncle had a stroke because of it, so I don’t miss the hypertension drugs”* (Male, 52 years; HTN [56]).

#### Use of alternative and complementary treatments.

Low confidence in NCD medications led many patients to supplement or replace treatment with herbal remedies included consuming bitter herbs and vegetables (such as ginger, garlic, *mululuza* (*Vernonia amygdalina*), prayer, or consulting with traditional healers [52,55,57,59,60]. The belief that these remedies would cure NCDs and positive experiences of symptom relief often reinforced trust in these remedies:

*“I take the mushroom when I see that my heart rate is high. … it helps with blood pressure. When I don’t have it; I know I’m putting my life at risk.* (Female, 60 years; HTN [57]).

#### Stress reduction techniques to manage HTN.

Some patients attributed HTN to emotional stress and adopted avoidance or withdrew strategies including avoiding conflicts, noisy environments, and stressful discussions to manage HTN [48,57,59,60].

*“I should not think a lot, …neither should someone speak to me in a loud frightening way”* (Female, 49 years; HTN [57]).

#### Managing multiple medications (Polypharmacy).

Patients adopted practical strategies to manage complex treatment regimens, including synchronizing medication schedules, storing medications together in “drug banks,” and involving family members in supporting treatment adherence [50,56,59]. However, concerns about pill burden and possible drug toxicity due to the many medicines led some to substitute them with physical exercises, herbal remedies, or to discontinue NCD medications [48,59]:

*“I refused taking medication for the virus and for pressure … that is too many drugs in your body”* (Female, 42 years; HTN [48]).

### Theme three: Patients adopting health-promoting behaviors

Patients engaged in a range of various non-pharmacological practices to manage HTN and DM, often based on health provider advice or self-initiation.

#### Dietary modifications and challenges.

Many patients attempted to change their diet by reducing salt, sugar, and fatty foods, eating smaller portions more frequently, drinking a lot of water to “flush out” medicines, and eating food before taking medicines to prevent side-effects [48,51,53,57,60]. However, food insecurity, alongside poverty, limited food options and social norms on food consumption undermined planned dietary changes and medicine adherence, particularly when medicines had to be taken with food [49–52]:

*“This is why I have to skip taking my insulin because my body gets weak, I shake and feel like going mad. … because I don’t have food”* (woman, 46-years, DM [52]).

#### Lifestyle adjustments and condition-specific practices.

In addition to dieting, patients engaged in physical activity, reduced alcohol intake, and avoiding smoking [49,51,53,60]. Some patients also took immune boosters to enhance their immunity. We also observed condition-specific practices in this population. For HIV, patients emphasized safe sex and wound care [53]. For HTN and DM, self-monitoring of blood pressure and glucose was highlighted, though glucose monitoring was often limited by cost [50,59]. For DM only, additional practice was foot care [53]:

*“… for type two diabetes, … proper foot care, included washing and drying feet properly; and wearing the right shoes “to be safe*” [53].

### Theme four: Coping with psychosocial distress

Patients used psychological and social strategies to manage the emotional burden of multimorbidity.

#### Acceptance as a coping mechanism.

Acceptance of illness, particularly HIV, emerged as a key psychological adaptation that enabled patients to rebuild self-worth and engage in self-care [50,53,54]. Patients described reaching acceptance through counseling and gradual emotional adjustment [51,53,54]:

*“I could not accept living with HIV, but as time went by, I accepted … and that’s what I am now”* (P1, Female, HTN [53]).

#### Managing stigma.

HIV-related stigma drove many patients to conceal their status and ART use, mostly through maintaining secrecy of status, hiding HIV medicines and social withdrawal [51,53,59]. In severe cases, some patients would physically withdraw from stigmatizing environments:

*“My brothers stigmatize me a lot. …, they insult me the whole night... So sometimes I don’t even stay at my own home.”* (Female, 44 years [59]).

#### Maladaptive coping.

Some patients resorted to maladaptive coping strategies [51,60], particularly alcohol use to cope with depression associated with multimorbidity [51]. While drinking alcohol offered temporary relief from the feelings of hopelessness, it risked worsening their health outcomes [51,60]:

*“I do drink alcohol, … Yes, I sometimes have that problem [depression] I drink alcohol day before going to clinic”* (Female, 48 years old; HTN [51]).

### Theme five: Strategies to navigate healthcare insecurities

Patients described navigating systemic inequities between HIV and NCD services [50,52–56,58,59]. While HIV services were well-structured and free, HTN and DM care were poorly integrated and under-resourced.

#### Navigating limited medicine availability.

The most recurrent challenge was the inconsistent availability of essential medicines for HTN and DM. Many patients were told to purchase medications out-of-pocket, which was financially unsustainable [50,52,53,55,56,58–60]. Consequently, some patients resorted to visiting multiple clinics to find medicines, borrowing from peers, substituting prescribed drugs with painkillers and in more constrained situations, patients discontinued treatment altogether until they could afford refills [50,52,56,60]:

*“They give you one pack and tell you to buy the rest. You may not have money for buying drugs at that time.”* (Woman, 42 years, DM [55]).

These shortages were reported across both fragmented and integrated HIV/NCD service models, underscoring persistent inequities between HIV and NCD care systems [49,50,52,55,56,58].

### Seeking multiple sources of care and information

To address the limited access to comprehensive care for HTN and DM, patients often sought services across multiple health facilities for consultations, expertise treatment and information [52,59,60]. This strategy was particularly common among patients managing DM [52,58]:

*“The problem is that one doctor will tell you to do this, and another asks you to do a different thing”* (woman, 46-years HTN, DM [58]).

### Financial coping strategies to sustain self-care

Lastly, to meet self-care costs, especially for NCD medications, transport, and recommended diets, patients employed a variety of income-generating or resource-mobilizing strategies. These included working extra hours, renting out their rooms, selling farm produce or household assets, and borrowing money to sustain care [50,52,55]:

*I always try to ensure that I collect wood to sell so I can go to the clinic. Or if I cannot find anyone to sell to, I borrow money, even if I do not know how I am going to return it*” (Female, 46 years; HTN [50]).

In some instances, some sought financial assistance from family, community, and church members to afford the cost of medication and consultations [60].

### Credibility assessment of review findings

We assessed the strength of evidence for each review finding using the CERQual approach and confidence ratings are shown in Table 4. Nineteen key summary review findings, each corresponding with the sub-themes, were created and assessed for confidence. Eleven of the 19 review findings were rated with “high confidence”; six with “moderate confidence”; and two with “low confidence”. The main reasons for low confidence were concerns with methodological limitations of contributing studies and inadequacy of data.

**Table 4:** GRADE-CERQUal assessment of summary findings.

*Summary of review findings*	Studies contributing to the review finding	CERQual assessment of confidence in evidence	Explanation of CERQual assessment *(NB: all studies from LMICs).*
***Theme 1:***			
* Ranking the severity of illnesses and efficacy of medicines: In their daily self-management, patients had a practice of grading the severity of each illness that lived with, and in most cases, they ranked HIV as a mild illness (and perceived HTN and DM comorbidities as severe and life threatening). They also ranked ART as “better” and efficacious, while NCD medicines perceived as less efficacious.	[48–52,56–60]	High confidence	Only one of the eight studies had a minor methodological limitation. No or minor concerns about coherence, relevance, and adequacy of data.
* Prioritizing care for the life-threatening comorbidities: Patients prioritized caring for HTN and DM. In their daily tasks, patients supplemented care for these NCD with eating various vegetables and herbs. However, care for these comorbidities was viewed as stressful due to the many tasks involved.	[48,49,51,56,58–60]	High confidence	One of the six studies had a minor methodological limitation. No or minor concerns about coherence, relevance, and adequacy of data.
***Theme 2:***			
* ART was the sole HIV treatment: patients used ART as the main treatment for HIV and perceived ART as highly efficacious and consistently adhered to it.	[49,50,52,54,56,57,59,60]	High confidence	One study with moderate methodological limitation. No or minor concerns about coherence, relevance, and adequacy of data.
* Intentional non-adherence to HTM and DM medication: Some patients were intentionally reluctant to take HTN or DM medications even when these medicines were available mostly due to doubts in their efficacy and side-effects.	[48,49,52,56–60]	High confidence	No or minor concerns about methodological limitation, coherence, relevance, and adequacy of data.
* Use of complementary treatments for the NCDs: Patients resorted to using traditional/herbal medicines including taking herbal concoctions; consuming bitter vegetables; drinking lots of water, spiritual interventions and visiting traditional healers.	[52,54,55,57,59,60]	Moderate confidence	Two studies with moderate methodological limitations. No or minor concerns about coherence, relevance, and adequacy of data.
* Use of stress reduction techniques: Since patients believed that stress is the cause of HTN, they employed techniques to reduce stress including avoiding stressful relationships, avoiding noisy environments, and not talking about experiences that trigger stress or worries.	[48,57,59]	Moderate confidence	One study with moderate concern about adequacy of data. No or minor concerns about methodological limitations, coherence, and relevance.
			
* Strategies to manage adhering to the different medicines: These included: swallowing all the types at the same time, keeping all the medicines in the same drug bag and involving family members to give them reminders.	[50,56,57,59]	High confidence	No or minor concerns about methodological limitation, coherence, relevance, and adequacy of data.
* Managing the pill burden: Patients felt the burden of taking many pills and some feared possible side effects of taking different medicines. Therefore, patients would sometimes pause taking NCD medicines and would substitute them with taking herbal treatments or doing physical exercises.	[48,59]	Moderate confidence	One study with moderate concern about adequacy of data. No or minor concerns about methodological limitations, coherence, and relevance.
**Theme 3:**			
* Patients embraced changes in feeding behavior: Patients changed feeding practices by reducing the in-take of salty, sugary and fatty foods, eating small portions of food frequently, and drinking plenty of water.	[51,53,57,59]	High confidence	One study with a minor methodological limitation. No or minor concerns about coherence, relevance, and adequacy of data.
* Patients resorted to eating any type of food which was available, including unhealthy food, due to inability to afford or difficulties in preparing different meals for their family members	[49,50,53,57,59]	High confidence	No or minor concerns about methodological limitation, coherence, relevance, and adequacy of data.
* Taking medicines after a meal: Patients mentioned taking medicines after eating food, which negatively affected their adherence to medicines when they lacked food.	[50–52]	Moderate confidence	Moderate concerns about adequacy with one study. No or minor concerns about methodological limitation, and coherence, relevance.
* Making healthy lifestyle choices: The lifestyle choices/ practices included engaging in physical exercises, quitting or reducing alcohol consumption, not smoking and some took immune boosters.	[49–53,57,60]	High confidence	One study with a mild methodological limitation. No or minor concerns about coherence, relevance, and adequacy of data.
* Disease-specific health promoting practices, which included: a) safe sex and wound protection (for HIV); b) blood pressure monitoring for HTN; c) blood glucose monitoring for DM; d) footcare and wound care for DM care	[50,53,59]	Low confidence	Moderate concerns about coherence and adequacy of data in the three studies. No or minor concerns about methodological limitation, and relevance.
**Theme 4:**			
* Accept to live with illnesses: Patients ensured to develop a positive attitude towards living with lifelong illnesses, especially with HIV.	[50,53,54]	Moderate confidence	One study with a moderate methodological limitation. No or minor concerns about coherence, relevance, and adequacy of data
* Strategies to deal with stigma: patients made the following to deal with stigma: being discreet about their condition, hiding their medicines, and staying away from their homes when they felt rejected.	[51,53,54,59]	Moderate confidence	One study with a moderate methodological limitation and another with mild methodological limitation. No or minor concerns about coherence, relevance, and adequacy of data.
* Coping with depression: In some cases, patients resorted to drinking alcohol to cope with depression resulting from living with multiple chronic illnesses.	[48,51]	Low confidence	Moderate concerns about methodological limitations and adequacy of data. No or minor concerns about coherence, relevance.
**Theme 5:**			
* Coping with unreliable availability of NCD medicines and supplies: this included Patients buying their own supplies out of pocket; visiting/ attending different facilities in search of medicines; Patients substituting unavailable medications with painkillers; borrowing medication and enduring to live without taking medicines and disease monitoring	[50,52,53,55,56,58–60]	High confidence	One study with a moderate methodological limitation. No or minor concerns about coherence, relevance, and adequacy of data.
* Personal efforts to improve access to healthcare and health information: This entailed active search for healthcare information, and attending multiple clinics for consultation, specific treatments.	[50,52,53,58–60]	High confidence	No or minor concerns about methodological limitation, coherence, relevance, and adequacy of data.
* Personal efforts to raise money for self-care. Practices include working extra hours for additional income; Borrowing money to meet medical bills; selling of personal items; and seeking money from social networks.	[50,53,55,57–60]	High confidence	One study with a moderate methodological limitation. No or minor concerns about coherence, relevance, and adequacy of data.

* = Summary of review finding.

### Reflection on treatment burden

Treatment burdens fell under the four domains of the NPT (coherence, cognitive participation, collective action, and reflexive monitoring) with an additional “unallocated” domain (Table 5). Under *coherence*, patients struggled with sense-making as they reconciled multiple illnesses, complex regimens, emotional distress and stigma. *Cognitive participation* highlighted patients’ efforts to engage in care through mobilizing family support, maintaining social relationships despite stigma, and navigating inconsistent provider communication. *Collective action* captured practical challenges in care, including polypharmacy and complex routines, side effects, lifestyle and dietary changes, and reliance on alternative therapies, all happening within under-resourced NCD-care. *Reflexive monitoring* illustrated how patients evaluated and adapted care practices in response to constraints, such as changing medication schedules and compromising diet. The *unallocated domain* captured cross-cutting financial strains and efforts to meet basic care needs, including securing money for food, medicines, and transport, which underpinned all other burdens.

**Table 5:** Treatment burden among People living with HIV and hypertension or diabetes mapped on the Normalization Process domains.

Treatment burden domain and mapped *Summary of review finding*	Corresponding treatment burden
**1. Coherence/ Sense making of HIV and chronic NCD comorbidities**	
* *Ranking of illnesses and medications:*	• Understanding each illness and its implications^a^
	• Interpreting and managing multiple medications^a^
	• Differentiating illness experiences between HIV and NCDs ^a^
* Prioritize care for the life-threatening comorbidities:	• Limited knowledge on managing multiple conditions ^b^
	• Taking on extra patient-hood tasks to manage NCDs ^c^
* Accept to live with illnesses:	• Psychological burden of living with lifelong conditions ^a^
	• Managing illness-related emotional distress^a^
	• Navigating internal stigma ^c^
**2. Cognitive Participation (Engaging with others)**	
* *Strategies to manage the different medicines:*	• Involving family in adherence ^a^
* *Strategies to deal with stigma:*	• Learning to cope with stigma ^a^
*	• Maintaining relationships despite stigma^a^
* *Personal efforts to improve access to healthcare and health information:*	• Poor communication with healthcare providers on HTN and DM ^b^.
	• Conflicting health worker advice on HTN/DM care ^b^
	• Building motivation for long-term self-care^a^
**3: Enacting management strategies**	
* ART was the sole HIV treatment:	• Maintaining daily ART routines ^a^
* Use of stress reduction techniques:	• Enacting stress relief practices to manage HTN ^c^
* Use of complementary treatments for NCDs:	• Use of traditional/herbal/spiritual treatments ^c^
* Taking medicines after a meal:	• Ensuring meals before medication ^a^
* Strategies to manage the different medicines:	• Swallowing multiple pills daily ^a^
* Managing the pill burden:	• Coping with pill burden ^a^
	• Coping with medication side effects ^a^
* Patients embraced changes in feeding behavior:	• Struggling to access or afford proper diet ^a^
	• Limited dietary knowledge ^b^
	• Difficulty preparing special meals in shared households ^a^
* Making healthy lifestyle choices:	• Limited access to lifestyle modification information ^b^
	• Attempting lifestyle changes with limited support ^a^
* Coping with unreliable availability of NCD medicines, supplies and health information	• Inadequate NCD support compared to HIV care^b^
	• Visiting multiple clinics for NCD care ^b^
	• Borrowing medicines^b^
	• Confusion from inconsistent and mixed messages on HTN/DM care^b^
**4: Reflecting on management**	
* Coping with unreliable availability of NCD medicines and supplies:	• Reassessing treatment options and switching regimens ^c^
	• Assessing value of care received for HTN/DM compared to HIV ^c^
	• Purchasing unavailable medicines or devices ^a^.
	• Unintentionally skipping NCD medications^a^.
* *Patients resorted to eating any type of food which was available*	• Enduring or compromising with unhealthy food options ^a^.
**5. Unallocated**	
* Personal efforts to raise money:	• Efforts to raise money for medicines, food and basic needs ^a,b^.

* = Summary of review finding.

HTN = hypertension, DM = diabetes mellitus; NCD = non-communicable disease; Treatment burden arising from: ^a^ = prescribed healthcare tasks; ^b^ = health system inequalities between HIV and NCDs; ^c^ = Patient-initiated practices. (Table adapted from Chikumbu et al., 2022 [28].

Overall, treatment burden arose from three sources: a) prescribed healthcare tasks (with most burdens); b) systemic inequalities between HIV and NCD services; and c) Patient-initiated strategies. Most burdens were related to healthcare tasks assigned by providers to manage the multiple conditions to compensate for gaps in care, including traditional therapies and stress-relief practices.

## Discussion

This review examined self-care practices and related treatment burdens among PLHIV with HTN or DM comorbidities in LMICs. Patients used both pharmacological practices (such as sense-making of illnesses and divergent treatment strategies) and non-pharmacological strategies (including lifestyle changes, psychosocial coping and navigating health systems) to manage HIV and NCDs concurrently. These findings add to the growing evidence that self-care for chronic conditions extends beyond medical management to include locally adapted practices for maintaining well-being [61–63]. Importantly, findings demonstrate that self-care practices for PLHIV and HTN and/or DM comorbidity are shaped by illness perceptions, structural constraints and the cumulative demands of managing multimorbidity, reflecting the influence of personal and environmental factors on self-care in the resource-limited settings [62].

Patients engaged consistently perceived HIV as more manageable than HTN or DM. Highlighting the success of long-standing HIV investments such as free ART, differentiated service delivery, peer support, and structured counselling that have improved trust, adherence, and retention in care [64,65]. In contrast, HTN and DM were viewed as life-threatening and therefore required greater attention, increasing the care burden associated with these NCDs, consistent with other studies in low-resource settings [66,67]. These perceptions likely reflect persistent gaps in NCD infrastructure, medicine availability, and patient support in many LMICs [68,69]. These findings therefore support calls to adapt successful HIV care strategies, such as decentralization, task-shifting and community engagement, within integrated HIV/NCD care models [70,71].

Adherence to medicines differed across conditions. While ART was consistently taken due to reliable access and strong confidence in its effectiveness, adherence to HTN and DM medications was often selective. Similar patterns of low adherence to NCD medication have been reported in resource-limited settings and are linked to asymptomatic disease, medicine side effects, inconsistent clinical advice, and financial barriers [60,63]. The use of herbal remedies, spiritual practices, or stress-relief strategies appeared to be adaptive responses to gaps in formal HTN and DM care and these remain common in African settings [72,73]. Leveraging existing HIV care platforms, integrated HIV/NCD services can strengthen health education, particularly medication counselling on disease progression, treatment benefits, side effects, and drug interactions, to improve treatment engagement, as demonstrated in successful HIV care across LMICs [74,75].

Lifestyle-related self-care practices, particularly dietary adjustments and physical activity, were commonly attempted but difficult to sustain due to poverty, food insecurity and limited social support, consistent with prior evidence in low-resource settings [76,77]. These findings suggest that lifestyle counselling, including dietary guidance, should be context-sensitive and linked to feasible local resources. Generic lifestyle advice without addressing structural constraints is unlikely to be effective [78].

Psychosocial coping emerged as an important but under-addressed component of self-care. While acceptance of illness and counseling supported emotional well-being, maladaptive strategies like secrecy about HIV status, social withdrawal, and alcohol use undermined adherence and health outcomes. HIV-related stigma is well-established as a barrier to treatment engagement in sub-Saharan Africa, driving ART concealment, missed appointments, and disengagement from care [79,80]. Moreover, noted alcohol use also poses significant physiological and pharmacological risks as it accelerates HIV, HTN and DM progression, reduces ART effectiveness and increases susceptibility to other conditions like liver disease [81]. These findings align with growing calls to embed mental health screening, peer-support networks, and structured stigma-reduction programs within integrated HIV/NCD care platforms [61,82], recognizing that psychosocial well-being is not ancillary to self-care but constitutive of it.

The review highlights substantial care burdens driven by disparities between HIV and HTN or DM services. Patients often attended multiple clinics, borrowed medicines, and generated additional income to sustain care, coping practices reported among people with NCDs in resource-limited settings [83,84]. These burdens were far less evident in HIV care, underscoring persistent inequities within the integrated service delivery [19,21]. Mapping findings onto the NPT further showed how self-care tasks accumulate into daily workload, consistent with evidence that multimorbidity increases care burden in constrained health systems [28,85]. Strengthening equitable integrated HIV/NCD care through expanding access to HTN and DM medicines, health information and patient support within the same service platforms could reduce patient workload.

### Strengths and limitations

This review provides a theory-informed synthesis of patient experiences across low-resource settings, offering transferable insights for integrated HIV/NCD care. However, included studies were geographically concentrated in Africa, which may limit transferability to other LMICs. This is because the health systems, HIV care infrastructure, NCD service availability and sociocultural contexts differ across LMIC regions and may influence the transferability of findings to Asia, Latin America, or other settings. Second, no eligible quantitative studies were identified. While qualitative studies provided rich insights into patients’ experiences, perceptions, and coping strategies, they did not allow estimation of the prevalence or magnitude of self-care practices or treatment burden. Findings should therefore be interpreted as reflecting patient experiences and perceptions rather than condition-specific or population-level estimates of self-care practices. Future quantitative research should complement these findings by quantifying self-care practices and measuring the magnitude and distribution of treatment burden among PLHIV with NCD comorbidities in LMIC settings. Publication bias may exist if studies reporting negative self-care experiences or failures in HIV/NCD care remain unpublished. Also, subgroup comparisons by age, country, or other demographic characteristics were not feasible due to inconsistent reporting across studies. Finally, treatment burdens were derived from self-reported qualitative data and may not fully capture their intensity or fluctuation over time.

### Recommendations for practice and research

Strengthening self-care among PLHIV care requires addressing key gaps in the NCD service delivery that drive patient workload. Firstly, ensuring a reliable and uninterrupted supply of essential HTN and DM medicines within HIV platforms will be critical for continuity of care and reducing treatment burden. In addition, structured medication counselling should be provided to address safe medicine use, potential drug interactions, side effects, and the risks associated with unregulated herbal remedies. Health education should explicitly address the perceived inefficacy on NCD medications and misconceptions about cure. Lifestyle guidance should be tailored to patients’ socioeconomic realities, recognizing that poverty and food insecurity often limit the feasibility of dietary advice and lifestyle recommendations. Where possible, dietary guidance should be linked to locally available resources, including community or food support programs. Furthermore, integrating psychosocial support and stigma-reduction interventions into HIV/NCD services is essential, as emotional distress, stigma, and maladaptive coping can undermine sustained engagement in care.

Future research should build on these findings by quantifying self-care workload and estimating the magnitude and distribution of treatment burden among PLHIV and NCD comorbidities in LMICs. Implementation studies are also needed to evaluate whether health system strategies, such as task-shifting, decentralized service delivery, and differentiated care models, can effectively reduce patient workload and improve long-term self-care outcomes..

## Conclusion

Self-care among PLHIV and comorbid HTN or DM in LMICs is shaped by illness perceptions, structural inequities, and the cumulative demands of multimorbidity. Self-care practices included making sense of illnesses and medications, prioritizing treatment across conditions, adopting lifestyle changes, coping with psychosocial distress, and navigating healthcare insecurities. These practices generated substantial treatment burdens arising from prescribed healthcare tasks, limited support for HTN and DM care, and patient efforts to bridge service gaps. Effective self-care therefore requires more than patient-level interventions. Strengthening integrated care through reliable access to NCD medicines, structured medication counselling, psychosocial support, and context-sensitive lifestyle guidance is essential to reduce patient workload and enable sustainable self-management. Future research should quantify self-care workload and examine how treatment burdens accumulate and interact to influence adherence, coping and health outcomes to inform more effective integrated care models.

## Supporting information

S1 Table PRISMA 2020 Checklists. (DOCX)

S2 Table ENTREQ Checklist. (DOCX)

S3 Table Search Strategy for databases. (DOCX)

S4 Table Excluded papers and reasons for exclusion. (DOCX)

S5 Table Analysis matrix table. (DOCX)
