# Supplementary material for: Self-care practices of people living with HIV and hypertension or diabetes comorbidities in low-and-middle income countries: A systematic review
Source: PLoS One. 2026 Oct 9;21(10):e0360076. doi: 10.1371/journal.pone.0360076 (PMC13652238; doi:10.1371/journal.pone.0360076)
Supplement: Supplementary file 3 [file pone.0360076.s003.docx]

**Supplementary Table 3: Search Strategy for some databases**

|  | **Table 3.1: Ovid MEDLINE** |  |
| --- | --- | --- |
| **#** | **Searches** | **Results**  **2013 to 2026** |
| 1 | (Self-care or selfcare or self-manage* or selfmanage* or self-monitor* or selfmonitor* or self efficacy or self-treat* or treat* or selftreat* or self help or Self-regulat* or Selfregulat* or adherence or self-medicat* or selfmedicat* or physical activity or physical exercise* or weight management or self-administrat* or selfadministrat* or self-test* or selftest*).mp. | 8816372 |
| 2 | exp Self Care/ | 68072 |
| 3 | 1 or 2 | 8816372 |
| 4 | (HIV or HIVAIDS or acquired immune deficiency syndrome or SIDA or AIDS or Human immunodeficiency virus* or immune failure or PLWHIV or PLHIV or HIV1 or HIV2).mp. | 558834 |
| 5 | exp HIV/ | 111969 |
| 6 | exp HIV Infections/ | 335888 |
| 7 | 4 or 5 or 6 | 568606 |
| 8 | (Hypertension* or high blood pressure* or HT or HTN or blood pressure* high or hyperpiesi*).mp. | 722941 |
| 9 | exp Hypertension/ | 337965 |
| 10 | 8 or 9 | 723086 |
| 11 | (Diabetes or high blood sugar or hyperglycemi* or DM or diabetic).mp. | 1042282 |
| 12 | exp Diabetes Mellitus/ | 567028 |
| 13 | glucose metabolism disorders/ or hyperglycemia/ or glucose intolerance/ | 45051 |
| 14 | 11 or 12 or 13 | 1048079 |
| 15 | 10 or 14 | 1627952 |
| 16 | 3 and 7 and 15 | 6631 |
| 17 | (afghan* or africa* or albania* or algeria* or angola* or antigua* or barbuda* or argentin* or armenia* or aruba* or azerbaijan* or bahrain* or bangladesh* or bengal* or bangal* or barbados* or barbadian* or bajan or bajans or belarus* or belorus* or byelarus* or byelorus* or belize* or benin* or dahomey or bhutan* or bolivia* or bosnia* or herzegovin* or botswan* or batswan* or bechuanaland* or brazil* or brasil* or bulgaria* or burkina* or burkinese* or upper volta* or burundi* or urundi* or cabo verde* or cape verde* or cambodia* or kampuchea* or khmer* or cameroon* or cameroun* or ubangi shari* or chad* or chile* or china* or chinese or colombia* or comoro* or comore* or comorian* or mayotte* or congo* or zaire* or costa rica* or "cote d'ivoir*" or "cote d' ivoir*" or cote divoir* or cote d ivoir* or ivory coast* or ivorian* or croatia* or cuba or cuban or cubans or "cuba's" or cyprus* or cypriot* or czech* or djibouti* or french somaliland* or dominica* or ecuador* or egypt* or united arab republic* or el salvador* or salvadoran* or guinea* or equatoguinea* or eritrea* or estonia* or eswatini* or swaziland* or swazi* or swati* or ethiopia* or fiji* or gabon* or gabonese* or gabonaise* or gambia* or ((georgia or georgian or georgians) not (atlanta or california or florida)) or ghana* or gibraltar* or greece* or greek* or grecian* or grenada* or grenadian* or guam* or guatemala* or guyana* or guiana* or guyanese* or haiti* or hispaniola* or hondura* or hungary* or hungarian* or india* or indonesia* or iran* or iraq* or isle of man* or jamaica* or jordan* or kazakh* or kenya* or karabati* or korea* or kosovo* or kosova* or kyrgyz* or kirgiz* or kirghiz* or laos or lao or laotian* or latvia* or lebanon* or lebanese* or lesotho* or lesothan* or lesothonian* or basutoland* or mosotho* or basotho* or liberia* or libya* or jamahiriya* or lithuania* or macedonia* or madagasca* or malagasy* or malawi* or nyasaland* or malaysia* or malay* federation or maldives* or maldivian* or indian ocean or mali or malian* or "mali's" or malta or maltese* or "malta's" or micronesia* or marshallese* or kiribati* or marshall island* or nauru or nauran or nauruans or "naurian's" or mariana or marianas or palau or paluan* or tuvalu* or mauritania* or mauritan* or mauritius* or mexico* or mexican* or moldova* or moldovia* or mongol* or montenegr* or morocco* or moroccan* or ifni or mozambique* or mozambican* or myanmar* or burma* or burmese or namibia* or nepal* or new caledonia* or netherlands antill* or nicaragua* or niger* or oman or omani or omanis or "oman's" or pakistan* or palestin* or gaza* or west bank* or panama* or paraguay* or peru or peruvian* or "peru's" or philippine* or philipine* or phillipine* or phillippine* or filipino* or filipina* or poland* or polish or pole or poles or portugal* or portuguese or puerto ric* or romania* or russia* or ussr* or soviet* or rwanda* or rwandese or ruanda* or ruandese or samoa* or navigator island* or pacific island* or polynesia* or "sao tome and principe*" or sao tomean* or santomean* or saudi arabia* or saudi or saudis or senegal* or serbia* or seychell* or sierra leone* or slovak* or sloven* or melanesia* or solomon island* or norfolk island* or somali* or sri lanka* or ceylon* or "saint kitts and nevis*" or "st kitts and nevis*" or kittian* or nevisian* or saint lucia* or st lucia* or saint vincent* or st vincent* or vincentian* or grenadine* or sudan* or surinam* or syria* or tajik* or tadjik* or tadzhik* or tanzania* or tanganyika* or thai* or timor leste* or east timor* or timorese* or togo or togoles* or "togo's" or tonga* or trinidad* or tobago* or tunisia* or turkiy* or turkey* or turk or turks or turkish or turkmen* or uganda* or ukrain* or uruguay* or uzbek* or vanuatu* or new hebrides* or venezuela* or vietnam* or viet nam* or yemen* or yugoslav* or zambia* or zimbabwe* or rhodesia* or arab* countr* or middle east* or global south or sahara* or subsahara* or magreb* or maghrib* or west indies* or caribbean* or central america* or latin america* or south america* or central asia* or north asia* or northern asia* or southeastern asia* or south eastern asia* or southeast asia* or south east asia* or west asia* or western asia* or east europe* or eastern europe* or developing countr* or developing nation* or developing population* or developing world or less developed countr* or less developed nation* or less developed world or lesser developed countr* or lesser developed nation* or lesser developed world or under developed countr* or under developed nation* or under developed world or underdeveloped countr* or underdeveloped nation* or underdeveloped world or middle income countr* or middle income nation* or middle income population* or low income countr* or low income nation* or low income population* or lower income countr* or lower income nation* or lower income population* or underserved countr* or underserved nation* or underserved population* or under served population* or under served nation* or under served population* or deprived countr* or deprived population* or high burden countr* or high burden nation* or countdown countr* or countdown nation* or poor countr* or poor nation* or poor population* or poor world or poorer countr* or poorer nation* or poorer population* or poorer world or developing econom* or less developed econom* or underdeveloped econom* or under developed econom* or middle income econom* or low income econom* or lower income econom* or low gdp or low gnp or low gross domestic or low gross national or lower gdp or lower gnp or lower gross domestic or lower gross national or lmic or lmics or third world or lami countr* or transitional countr* or emerging econom* or emerging nation*).ti,ab,hw,kf. | 3804076 |
| 18 | 16 and 17 | 2147 |
| 19 | limit 18 to (humans and yr="2013 -Current") | 1246 |
| 20 | limit 19 to ("young adult and adult (19-24 and 19-44)" or "middle aged (45 plus years)") | 880 |
|  |  |  |
|  | **Table 3.2: Embase Classic+Embase** |  |
| **#** | **Searches** |  |
| 1 | (Self-care or selfcare or self-manage* or selfmanage* or self-monitor* or selfmonitor* or self efficacy or self-treat* or treat* or selftreat* or self help or Self-regulat* or Selfregulat* or adherence or self-medicat* or selfmedicat* or physical activity or physical exercise* or weight management or self-administrat* or selfadministrat* or self-test* or selftest*).mp. | 13443849 |
| 2 | exp Self Care/ | 131530 |
| 3 | 1 or 2 | 13443849 |
| 4 | (HIV or HIVAIDS or acquired immune deficiency syndrome or SIDA or AIDS or Human immunodeficiency virus* or immune failure or PLWHIV or PLHIV or HIV1 or HIV2).mp. | 784399 |
| 5 | exp HIV/ | 237205 |
| 6 | exp HIV Infections/ | 941792 |
| 7 | 4 or 5 or 6 | 1238735 |
| 8 | (Hypertension* or high blood pressure* or HT or HTN or blood pressure* high or hyperpiesi*).mp. | 1558842 |
| 9 | exp Hypertension/ | 1277828 |
| 10 | 8 or 9 | 1718880 |
| 11 | (Diabetes or high blood sugar or hyperglycemi* or DM or diabetic).mp. | 2227239 |
| 12 | exp Diabetes Mellitus/ | 1599284 |
| 13 | glucose metabolism disorders/ or hyperglycemia/ or glucose intolerance/ | 172861 |
| 14 | 11 or 12 or 13 | 2247099 |
| 15 | 10 or 14 | 3448348 |
| 16 | 3 and 7 and 15 | 46482 |
| 17 | (afghan* or africa* or albania* or algeria* or angola* or antigua* or barbuda* or argentin* or armenia* or aruba* or azerbaijan* or bahrain* or bangladesh* or bengal* or bangal* or barbados* or barbadian* or bajan or bajans or belarus* or belorus* or byelarus* or byelorus* or belize* or benin* or dahomey or bhutan* or bolivia* or bosnia* or herzegovin* or botswan* or batswan* or bechuanaland* or brazil* or brasil* or bulgaria* or burkina* or burkinese* or upper volta* or burundi* or urundi* or cabo verde* or cape verde* or cambodia* or kampuchea* or khmer* or cameroon* or cameroun* or ubangi shari* or chad* or chile* or china* or chinese or colombia* or comoro* or comore* or comorian* or mayotte* or congo* or zaire* or costa rica* or "cote d'ivoir*" or "cote d' ivoir*" or cote divoir* or cote d ivoir* or ivory coast* or ivorian* or croatia* or cuba or cuban or cubans or "cuba's" or cyprus* or cypriot* or czech* or djibouti* or french somaliland* or dominica* or ecuador* or egypt* or united arab republic* or el salvador* or salvadoran* or guinea* or equatoguinea* or eritrea* or estonia* or eswatini* or swaziland* or swazi* or swati* or ethiopia* or fiji* or gabon* or gabonese* or gabonaise* or gambia* or ((georgia or georgian or georgians) not (atlanta or california or florida)) or ghana* or gibraltar* or greece* or greek* or grecian* or grenada* or grenadian* or guam* or guatemala* or guyana* or guiana* or guyanese* or haiti* or hispaniola* or hondura* or hungary* or hungarian* or india* or indonesia* or iran* or iraq* or isle of man* or jamaica* or jordan* or kazakh* or kenya* or karabati* or korea* or kosovo* or kosova* or kyrgyz* or kirgiz* or kirghiz* or laos or lao or laotian* or latvia* or lebanon* or lebanese* or lesotho* or lesothan* or lesothonian* or basutoland* or mosotho* or basotho* or liberia* or libya* or jamahiriya* or lithuania* or macedonia* or madagasca* or malagasy* or malawi* or nyasaland* or malaysia* or malay* federation or maldives* or maldivian* or indian ocean or mali or malian* or "mali's" or malta or maltese* or "malta's" or micronesia* or marshallese* or kiribati* or marshall island* or nauru or nauran or nauruans or "naurian's" or mariana or marianas or palau or paluan* or tuvalu* or mauritania* or mauritan* or mauritius* or mexico* or mexican* or moldova* or moldovia* or mongol* or montenegr* or morocco* or moroccan* or ifni or mozambique* or mozambican* or myanmar* or burma* or burmese or namibia* or nepal* or new caledonia* or netherlands antill* or nicaragua* or niger* or oman or omani or omanis or "oman's" or pakistan* or palestin* or gaza* or west bank* or panama* or paraguay* or peru or peruvian* or "peru's" or philippine* or philipine* or phillipine* or phillippine* or filipino* or filipina* or poland* or polish or pole or poles or portugal* or portuguese or puerto ric* or romania* or russia* or ussr* or soviet* or rwanda* or rwandese or ruanda* or ruandese or samoa* or navigator island* or pacific island* or polynesia* or "sao tome and principe*" or sao tomean* or santomean* or saudi arabia* or saudi or saudis or senegal* or serbia* or seychell* or sierra leone* or slovak* or sloven* or melanesia* or solomon island* or norfolk island* or somali* or sri lanka* or ceylon* or "saint kitts and nevis*" or "st kitts and nevis*" or kittian* or nevisian* or saint lucia* or st lucia* or saint vincent* or st vincent* or vincentian* or grenadine* or sudan* or surinam* or syria* or tajik* or tadjik* or tadzhik* or tanzania* or tanganyika* or thai* or timor leste* or east timor* or timorese* or togo or togoles* or "togo's" or tonga* or trinidad* or tobago* or tunisia* or turkiy* or turkey* or turk or turks or turkish or turkmen* or uganda* or ukrain* or uruguay* or uzbek* or vanuatu* or new hebrides* or venezuela* or vietnam* or viet nam* or yemen* or yugoslav* or zambia* or zimbabwe* or rhodesia* or arab* countr* or middle east* or global south or sahara* or subsahara* or magreb* or maghrib* or west indies* or caribbean* or central america* or latin america* or south america* or central asia* or north asia* or northern asia* or southeastern asia* or south eastern asia* or southeast asia* or south east asia* or west asia* or western asia* or east europe* or eastern europe* or developing countr* or developing nation* or developing population* or developing world or less developed countr* or less developed nation* or less developed world or lesser developed countr* or lesser developed nation* or lesser developed world or under developed countr* or under developed nation* or under developed world or underdeveloped countr* or underdeveloped nation* or underdeveloped world or middle income countr* or middle income nation* or middle income population* or low income countr* or low income nation* or low income population* or lower income countr* or lower income nation* or lower income population* or underserved countr* or underserved nation* or underserved population* or under served population* or under served nation* or under served population* or deprived countr* or deprived population* or high burden countr* or high burden nation* or countdown countr* or countdown nation* or poor countr* or poor nation* or poor population* or poor world or poorer countr* or poorer nation* or poorer population* or poorer world or developing econom* or less developed econom* or underdeveloped econom* or under developed econom* or middle income econom* or low income econom* or lower income econom* or low gdp or low gnp or low gross domestic or low gross national or lower gdp or lower gnp or lower gross domestic or lower gross national or lmic or lmics or third world or lami countr* or transitional countr* or emerging econom* or emerging nation*).ti,ab,hw,kf. | 4970416 |
| 18 | 16 and 17 | 12973 |
| 19 | limit 18 to (humans and yr="2013 -Current") | 9688 |
| 20 | limit 19 to (adult <18 to 64 years> or aged <65+ years>) | 4378 |
|  |  |  |
|  | **Table 3.3: APA PsycInfo** |  |
| **#** | **Searches** |  |
| 1 | (Self-care or selfcare or self-manage* or selfmanage* or self-monitor* or selfmonitor* or self efficacy or self-treat* or treat* or selftreat* or self help or Self-regulat* or Selfregulat* or adherence or self-medicat* or selfmedicat* or physical activity or physical exercise* or weight management or self-administrat* or selfadministrat* or self-test* or selftest*).mp. | 1189008 |
| 2 | exp Self-Care/ | 4909 |
| 3 | 1 or 2 | 1189008 |
| 4 | (HIV or HIVAIDS or acquired immune deficiency syndrome or SIDA or AIDS or Human immunodeficiency virus* or immune failure or PLWHIV or PLHIV or HIV1 or HIV2).mp. | 92886 |
| 5 | exp HIV/ | 52380 |
| 6 | HIV Infections/ | 0 |
| 7 | 4 or 5 or 6 | 92886 |
| 8 | (Hypertension* or high blood pressure* or HT or HTN or blood pressure* high or hyperpiesi*).mp. | 37973 |
| 9 | exp Hypertension/ | 9541 |
| 10 | 8 or 9 | 37973 |
| 11 | (Diabetes or high blood sugar or hyperglycemi* or DM or diabetic).mp. | 45356 |
| 12 | exp Diabetes Mellitus/ | 11709 |
| 13 | glucose metabolism disorders/ or hyperglycemia/ or glucose intolerance/ | 686 |
| 14 | 11 or 12 or 13 | 45356 |
| 15 | 10 or 14 | 75660 |
| 16 | 3 and 7 and 15 | 648 |
| 17 | (afghan* or africa* or albania* or algeria* or angola* or antigua* or barbuda* or argentin* or armenia* or aruba* or azerbaijan* or bahrain* or bangladesh* or bengal* or bangal* or barbados* or barbadian* or bajan or bajans or belarus* or belorus* or byelarus* or byelorus* or belize* or benin* or dahomey or bhutan* or bolivia* or bosnia* or herzegovin* or botswan* or batswan* or bechuanaland* or brazil* or brasil* or bulgaria* or burkina* or burkinese* or upper volta* or burundi* or urundi* or cabo verde* or cape verde* or cambodia* or kampuchea* or khmer* or cameroon* or cameroun* or ubangi shari* or chad* or chile* or china* or chinese or colombia* or comoro* or comore* or comorian* or mayotte* or congo* or zaire* or costa rica* or "cote d'ivoir*" or "cote d' ivoir*" or cote divoir* or cote d ivoir* or ivory coast* or ivorian* or croatia* or cuba or cuban or cubans or "cuba's" or cyprus* or cypriot* or czech* or djibouti* or french somaliland* or dominica* or ecuador* or egypt* or united arab republic* or el salvador* or salvadoran* or guinea* or equatoguinea* or eritrea* or estonia* or eswatini* or swaziland* or swazi* or swati* or ethiopia* or fiji* or gabon* or gabonese* or gabonaise* or gambia* or ((georgia or georgian or georgians) not (atlanta or california or florida)) or ghana* or gibraltar* or greece* or greek* or grecian* or grenada* or grenadian* or guam* or guatemala* or guyana* or guiana* or guyanese* or haiti* or hispaniola* or hondura* or hungary* or hungarian* or india* or indonesia* or iran* or iraq* or isle of man* or jamaica* or jordan* or kazakh* or kenya* or karabati* or korea* or kosovo* or kosova* or kyrgyz* or kirgiz* or kirghiz* or laos or lao or laotian* or latvia* or lebanon* or lebanese* or lesotho* or lesothan* or lesothonian* or basutoland* or mosotho* or basotho* or liberia* or libya* or jamahiriya* or lithuania* or macedonia* or madagasca* or malagasy* or malawi* or nyasaland* or malaysia* or malay* federation or maldives* or maldivian* or indian ocean or mali or malian* or "mali's" or malta or maltese* or "malta's" or micronesia* or marshallese* or kiribati* or marshall island* or nauru or nauran or nauruans or "naurian's" or mariana or marianas or palau or paluan* or tuvalu* or mauritania* or mauritan* or mauritius* or mexico* or mexican* or moldova* or moldovia* or mongol* or montenegr* or morocco* or moroccan* or ifni or mozambique* or mozambican* or myanmar* or burma* or burmese or namibia* or nepal* or new caledonia* or netherlands antill* or nicaragua* or niger* or oman or omani or omanis or "oman's" or pakistan* or palestin* or gaza* or west bank* or panama* or paraguay* or peru or peruvian* or "peru's" or philippine* or philipine* or phillipine* or phillippine* or filipino* or filipina* or poland* or polish or pole or poles or portugal* or portuguese or puerto ric* or romania* or russia* or ussr* or soviet* or rwanda* or rwandese or ruanda* or ruandese or samoa* or navigator island* or pacific island* or polynesia* or "sao tome and principe*" or sao tomean* or santomean* or saudi arabia* or saudi or saudis or senegal* or serbia* or seychell* or sierra leone* or slovak* or sloven* or melanesia* or solomon island* or norfolk island* or somali* or sri lanka* or ceylon* or "saint kitts and nevis*" or "st kitts and nevis*" or kittian* or nevisian* or saint lucia* or st lucia* or saint vincent* or st vincent* or vincentian* or grenadine* or sudan* or surinam* or syria* or tajik* or tadjik* or tadzhik* or tanzania* or tanganyika* or thai* or timor leste* or east timor* or timorese* or togo or togoles* or "togo's" or tonga* or trinidad* or tobago* or tunisia* or turkiy* or turkey* or turk or turks or turkish or turkmen* or uganda* or ukrain* or uruguay* or uzbek* or vanuatu* or new hebrides* or venezuela* or vietnam* or viet nam* or yemen* or yugoslav* or zambia* or zimbabwe* or rhodesia* or arab* countr* or middle east* or global south or sahara* or subsahara* or magreb* or maghrib* or west indies* or caribbean* or central america* or latin america* or south america* or central asia* or north asia* or northern asia* or southeastern asia* or south eastern asia* or southeast asia* or south east asia* or west asia* or western asia* or east europe* or eastern europe* or developing countr* or developing nation* or developing population* or developing world or less developed countr* or less developed nation* or less developed world or lesser developed countr* or lesser developed nation* or lesser developed world or under developed countr* or under developed nation* or under developed world or underdeveloped countr* or underdeveloped nation* or underdeveloped world or middle income countr* or middle income nation* or middle income population* or low income countr* or low income nation* or low income population* or lower income countr* or lower income nation* or lower income population* or underserved countr* or underserved nation* or underserved population* or under served population* or under served nation* or under served population* or deprived countr* or deprived population* or high burden countr* or high burden nation* or countdown countr* or countdown nation* or poor countr* or poor nation* or poor population* or poor world or poorer countr* or poorer nation* or poorer population* or poorer world or developing econom* or less developed econom* or underdeveloped econom* or under developed econom* or middle income econom* or low income econom* or lower income econom* or low gdp or low gnp or low gross domestic or low gross national or lower gdp or lower gnp or lower gross domestic or lower gross national or lmic or lmics or third world or lami countr* or transitional countr* or emerging econom* or emerging nation*).mp. [mp=title, abstract, heading word, table of contents, key concepts, original title, tests & measures, mesh word] | 661347 |
| 18 | 16 and 17 | 163 |
| 19 | limit 18 to (all journals and yr="2013 -Current") | 66 |
| 20 | limit 19 to human | 63 |
| 21 | limit 20 to "adulthood" | 49 |

| **Table 3.4** **AJOL manual search** | | |
| --- | --- | --- |
|  |  |  |
| Steps for the manual search | Description | Details |
| Database | Source of search | African Journals Online (AJOL) |
| 1. identify search terms | Keywords. Example: (HIV, OR PLHIV OR ART) AND ("hypertension" OR "diabetes") AND ("self-care" OR "self management" OR "qualitative") | Following the review search strategy. |
| 2. Search approach | Manual search via AJOL search bar with search terms including HIV, PLHIV AND hypertension" OR "diabetes AND self-care" OR "self management" OR  "Self monitoring" OR adherence | Searched using each keyword in AJOL search bar |
| 3. Initial title/abstract screening | Scan titles and abstracts to remove clearly irrelevant studies | - Excluded editorials, commentaries, reviews, letters, and irrelevant topics, which do not focus on people with HIV **and** hypertension/diabetes.    - Included primary studies conducted in LMICs, published between January 2013 and March 2026, regardless of study design or data collection method. studies |
| 4. Export titles/abstracts to Excel | Results manually copied into Excel for screening. (AJOL does not have a direct export function) | 45 records/ titles were exported |
| 5. Deduplication | Remove duplicate records: Duplicate titles or records across AJOL and other databases were identified and removed using Excel/ manual review. | 11 duplicates removed |
| 6. Identify unique titles from AJOL | After running duplicates, only titles from AJOL filtered for further screening | 28 titles and abstracts screened for eligibility |
| 7. Screening: title/abstract | Title and abstract screening conducted following the study criteria. | None of the 28 studies included: (For six studies, the outcomes were not related to selfcare, and 22 had a wrong population (non-HIV and hypertension or diabetes population)). |
