# Supplementary material for: Self-care practices of people living with HIV and hypertension or diabetes comorbidities in low-and-middle income countries: A systematic review
Source: PLoS One. 2026 Oct 9;21(10):e0360076. doi: 10.1371/journal.pone.0360076 (PMC13652238; doi:10.1371/journal.pone.0360076)
Supplement: Supplementary file 4 [file pone.0360076.s004.docx]

**Supplementary Table 4: Excluded papers and reasons for exclusion**

| **#** | **Study ID** | **Title** | **Reason for exclusion** |
| --- | --- | --- | --- |
| 1 | Murphy (2018) (1) | Hiv and Type 2 Diabetes (T2dm): A Qualitative Exploration of the Burden of Care Experienced and Perceived by Persons Living with the Comorbidities | Conference Issue/ paper |
| 2 | Manavalan (2019)(2) | Hypertension prevalence, awareness and barriers to control among persons living with HIV in northern Tanzania: A mixed methods study | Conference Issue/ paper |
| 3 | Greaves (2018)(3) | HIV and Type 2 diabetes: A qualitative exploration of the burden of care experienced and perceived by persons living with multiple co-morbidities in Barbados and Trinidad and Tobago | Conference Issue/ paper |
| 4 | Monroe (2013)(4) | Medication adherence in HIV-positive patients with diabetes or hypertension: a focus group study | Not in LMIC |
| 5 | Monroe (2018)(5) | Randomized controlled trial of a pictorial aid intervention for medication adherence among HIV-positive patients with comorbid diabetes or hypertension | Not in LMIC: in the USA/ John Hopkins |
| 6 | Tokwe (2021)(6) | Nursing Recommendations for the Management of HIV and Hypertension in a rural Primary Health Care Setting, Eastern Cape, South Africa | Wrong outcome - Recommendations to healthcare workers |
| 7 | Godongwana (2021)(7) | The comorbidity of HIV, hypertension and diabetes: a qualitative study exploring the challenges faced by healthcare providers and patients in selected urban and rural health facilities where the ICDM model is implemented in South Africa | Wrong outcome: focus on challenges |
| 8 | Melaku (2020)(8) | Glycemic control among people living with diabetes and Human Immunodeficiency Virus in Ethiopia: Leveraging Clinical Care for the looming Co-epidemics | Wrong outcome: glycemic control |
| 9 | Kagaruki (2018)(9) | Knowledge and perception on type2 diabetes and hypertension among HIV clients utilizing care and treatment services: a cross sectional study from Mbeya and Dar es Salaam regions in Tanzania | Wrong outcome: Not related to self-care |
| 10 | Chang (2019)(10) | Multimorbidity and care for hypertension, diabetes and hiv among older adults in rural South Africa | Wrong outcome: outcome is continuum of care |
| 11 | Palai (2019)(11) | Hypertension control in HIV-infected individuals at Princess Marina Hospital Infectious Disease Care Clinic | Wrong outcome: outcome is hypertension control |
| 12 | Bosire (2021)(12) | Patient-centred care for patients with diabetes and HIV at a public tertiary hospital in South Africa: an ethnographic study | wrong population - among healthcare providers |
| 13 | Mendenhall (2015)(13) | When HIV is ordinary and diabetes new: Remaking suffering in a South African Township | wrong population: DM only participants |
| 14 | Kendall (2018)(14) | Patient activation among people living with HIV: a cross-sectional comparative analysis with people living with diabetes mellitus | wrong population: HIV only, DM only participants (Single condition) |
| 15 | Mwangome (2016)(15) | I don't have options but to persevere." Experiences and practices of care for HIV and diabetes in rural Tanzania: A qualitative study of patients and family caregivers | Wrong population: HIV only, DM only participants (Single condition) |
| 16 | Badacho (2024) (16) | Lived experiences of people living with HIV and hypertension or diabetes access to care in Ethiopia: a phenomenological study | Wrong outcome: focus on access hypertension and diabetes care in southern Ethiopia. |
| 17 | Rumbidzai  (2024)(17) | Barriers and facilitators to primary care for people living with HIV and diabetes in Harare | Wrong outcome: focus on barriers and facilitators to the provision of care. |
| 18 | Manyazewal (2024) (18) | Digital continuous glucose monitoring systems for patients with HIV-diabetes comorbidity in Ethiopia: a situational analysis | Wrong outcome: Focus on utilization of Digital continuous glucose monitoring (CGM) systems |
| 19 | Maruma (2025)(19) | Exploring barriers to hypertension medication adherence among people living with HIV enrolled in the IHEART-SA trial: a theoretical domains framework qualitative study | Wrong outcome: focus on barriers and facilitators to care |
| 20 | Mhlanga (2024) (20) | Interventions for type 2 diabetes reduction among older people living with HIV in Harare | Wrong population: HIV only. |
| 21 | Ogugu (2024)(21) | An Examination of Contextual and Self-Management Factors in Relation to Blood Pressure Control among Adults Managing Comorbid HIV and Hypertension in Malawi | Meeting Issue/ abstract |
| 22 | Nang (2025) (22) | Barriers and facilitators of hypertension care among PLHIV at Yaounde Central Hospital, Cameroon. qualitative research 2024 | Wrong outcome: on barriers and facilitators to care |
| 23 | Akumu (2026) (23) | Diabetes mellitus, TB, and HIV multi-morbidities among adults in Uganda | Wrong outcome: Focus on prevalence of diabetes among adults with TB and HIV |

**REFERENCES**

1.	Murphy MM, Greaves NS, Pooransingh S, Samuels TA. Hiv and Type 2 Diabetes (T2dm): A Qualitative Exploration of the Burden of Care Experienced and Perceived by Persons Living with the Comorbidities. Journal of Epidemiology and Community Health. 2018;Conference:62nd Annual Scientific Meeting of the Society for Social Medicine. Glasgow United Kingdom. 72(Supplement 1) (pp A53).

2.	Manavalan P, Madut D, Thielman N, Okeke NL, Mmbaga B, Watt MH. Hypertension prevalence, awareness and barriers to control among persons living with HIV in northern Tanzania: A mixed methods study. Antiviral Therapy. 2019;Conference:21st International Workshop on Co-morbidities and Adverse Drug Reactions in HIV. Basel Switzerland. 4(Supplement 1) (pp A66).

3.	Greaves NS, Pooransingh S, Samuels TA, Murphy MM. HIV and Type 2 diabetes: A qualitative exploration of the burden of care experienced and perceived by persons living with multiple co-morbidities in Barbados and Trinidad and Tobago. West Indian Medical Journal. 2018;Conference:63rd Annual Caribbean Public Health Agency Research Conference, CARPHA 2018. St Kitts Marriott Resort and Royal Beach Casino Saint Kitts and Nevis. 67(Supplement 2) (pp 38).

4.	Monroe AK, Rowe TL, Moore RD, Chander G. Medication adherence in HIV-positive patients with diabetes or hypertension: a focus group study. Bmc Health Services Research. 2013;13.

5.	Monroe A, Pena J, Moore R, Riekert K, ... Randomized controlled trial of a pictorial aid intervention for medication adherence among HIV-positive patients with comorbid diabetes or hypertension. AIDS care. 2018.

6.	Tokwe L, Naidoo J. Nursing Recommendations for the Management of HIV and Hypertension in a rural Primary Health Care Setting, Eastern Cape, South Africa. Africa Journal of Nursing and …. 2021.

7.	Godongwana M, De Wet-Billings N, Milovanovic M. The comorbidity of HIV, hypertension and diabetes: a qualitative study exploring the challenges faced by healthcare providers and patients in selected urban and rural health facilities where the ICDM model is implemented in South Africa. BMC health services research. 2021;21(1).

8.	Melaku T, Chelkeba L, Mekonnen Z, ... Glycemic control among people living with diabetes and Human Immunodeficiency Virus in Ethiopia: Leveraging Clinical Care for the looming Co-epidemics. … Syndrome and Obesity. 2020.

9.	Kagaruki GB, Mayige MT, Ngadaya ES, Kilale AM, Kahwa A, Shao AF, et al. Knowledge and perception on type2 diabetes and hypertension among HIV clients utilizing care and treatment services: a cross sectional study from Mbeya and Dar es Salaam regions in Tanzania. BMC public health. 2018;18(1).

10.	Chang AY, Gomez-Olive FX, Manne-Goehler J, Wade AN, Tollman S, Gaziano TA, et al. Multimorbidity and care for hypertension, diabetes and hiv among older adults in rural South Africa. Bulletin of the World Health Organization. 2019;97(1):10-23.

11.	Palai T, Mosepele M, Mwita J. Hypertension control in HIV-infected individuals at Princess Marina Hospital Infectious Disease Care Clinic. Antiviral Therapy. 2019;Conference:21st International Workshop on Co-morbidities and Adverse Drug Reactions in HIV. Basel Switzerland. 4(Supplement 1) (pp A67).

12.	Bosire EN, Mendenhall E, Norris SA, Goudge J. Patient-centred care for patients with diabetes and HIV at a public tertiary hospital in South Africa: an ethnographic study. International Journal of Health Policy and Management. 2021;10(9):534-45.

13.	Mendenhall E, Norris SA. When HIV is ordinary and diabetes new: Remaking suffering in a South African Township. Global Public Health. 2015;10(4):449-62.

14.	Kendall CE, Shoemaker ES, Crowe L, MacPherson P, Becker ML, Levreault E, et al. Patient activation among people living with HIV: a cross-sectional comparative analysis with people living with diabetes mellitus. Aids Care-Psychological and Socio-Medical Aspects of Aids/Hiv. 2018;30(11):1444-51.

15.	Mwangome MN, Geubbels E, Klatser P, Dieleman M. I don't have options but to persevere." Experiences and practices of care for HIV and diabetes in rural Tanzania: A qualitative study of patients and family caregivers. International Journal for Equity in Health. 2016;15(1).

16.	Badacho AS, Mahomed OH. Lived experiences of people living with HIV and hypertension or diabetes access to care in Ethiopia: a phenomenological study. BMJ Open. 2024;14(2).

17.	Chireshe R, Naidoo K, Manyangadze T. Barriers and facilitators to primary care for people living with HIV and diabetes in Harare. African Journal of Primary Health Care and Family Medicine. 2024;16(a4603).

18.	Manyazewal T, Ali MK, Kebede T, Solomon S, Hailemariam D, Patel SA, et al. Digital continuous glucose monitoring systems for patients with HIV-diabetes comorbidity in Ethiopia: a situational analysis. Scientific Reports. 2024;14(11).

19.	Maruma W, Oladimeji E, Nyatela A, Heine M, Lalla-Edward ST, Venter WDF, et al. Exploring barriers to hypertension medication adherence among people living with HIV enrolled in the IHEART-SA trial: a theoretical domains framework qualitative study. Improving social inequities in health in the Global South. 2025;26(125).

20.	Mhlanga NL, Netangaheni TR. Interventions for type 2 diabetes reduction among older people living with HIV in Harare. South African Family Practice. 2024;66(1 Part 2).

21.	Ogugu E, Bidwell J, Butterfield R, Ruark A, Weiser S, Neilands T, et al. An Examination of Contextual and Self-Management Factors in Relation to Blood Pressure Control among Adults Managing Comorbid HIV and Hypertension in Malawi. Circulation. 2024;150(Supplement 1).

22.	Nang FDN, Mfeukeu LK, Ifoue RM, Kengne AP, Cheubo PJ, Tchitetchoun JPJ, et al. Barriers and facilitators of hypertension care among PLHIV at Yaounde Central Hospital, Cameroon. qualitative research 2024. Journal of the International Association of Providers of AIDS Care. 2025;24(23259582251383981).

23.	Akumu JE, Sekaggya-Wiltshire C, Babirye S, Musaazi J, Kukundakwe PE, Okiira C, et al. Diabetes mellitus, TB, and HIV multi-morbidities among adults in Uganda. Public Health Action. 2026;16(1):63-8.
