# Supplementary material for: Self-care practices of people living with HIV and hypertension or diabetes comorbidities in low-and-middle income countries: A systematic review
Source: PLoS One. 2026 Oct 9;21(10):e0360076. doi: 10.1371/journal.pone.0360076 (PMC13652238; doi:10.1371/journal.pone.0360076)
Supplement: Supplementary file 5 [file pone.0360076.s005.docx]

**Supplementary Table 5: Analysis matrix showing the analytical themes, contributing studies per sub-theme, descriptive themes/practices and corresponding quotations for the selfcare practices among PLHIV and hypertension or diabetes in LMICs.**

| **Themes** | **Sub themes** | **Contributing studies** | **Descriptive themes/**  **Practices** | **Sample Quotations / illustration from the literature** | **Study / Source of quotation** |
| --- | --- | --- | --- | --- | --- |
| **1. Patients made sense of their illnesses and medications by grading and prioritizing them** | Grading and classifying the severity of illnesses | (1-9) | Grading the illnesses: HIV ranked as less severe | But I saw that by looking, HIV is better than pressure that I have because with pressure, it does not go down or anything | Tokwe and Naidoo, 2020, pg6 |
|  |  |  |  | BP is dangerous because if people have not gone to the hospital, they may suffer a stroke or lose the use of some limbs or even die. Sometimes we hear of people just dying without getting sick. (male, 58y, 13 years) | Moucheraud et al., 2022, pg6 |
|  |  |  |  | Hypertension was believed to be more dangerous than HIV because it could lead to instantaneous death …  “I think it’s better to have HIV than pressure.... You know with HIV you can just take medication, and you will be fine. You can just continue with your life. But with pressure it can just go up suddenly and you can die instantly.” (Female, 44 years) | Manavalan et al., 2020, pg7 |
|  |  |  |  | All participants were asked to directly compare disease severity, and nearly half (45%) perceived hypertension as more severe than HIV; an additional 18% perceived the two diseases as equally severe. A common characterization of the diseases emerged that juxtaposed the experience of ‘dying instantly’ with hypertension against ‘being sick for a long time’ with HIV. "Hypertension can kill you tomorrow but HIV gives you time. Some diseases take life slowly while others take life instantly" (Male, 61 years old).  "The dangerous disease nowadays is blood pressure, because even sitting on this chair right now I can die. While HIV, you stay longer, you cannot die a sudden death. You have prolonged sickness for some days but not with high blood pressure" (Female, 70 years old). | Hing et al., 2019, |
|  |  |  |  | Participants found care for HTN/DM time-consuming and frustrating. | Ottaru et al., 2024 |
|  |  |  |  | Diabetes is considered a serious, life-threatening disease. Most of the participants.  It wasn’t easy; it wasn’t easy because my morale was at zero. HIV and diabetes, my morale was at zero, I told myself I was near the end. | Koffi et al., 2025. |
|  |  |  |  | Respondents with diabetes felt the most burdened, as self-managing diabetes required dietary and lifestyle changes, including strictly monitoring their sugar intake and required taking food with medication. | Van Pinxteren et al., 2023, pg4 |
|  | Prioritizing care for HTN and DM comorbidities | (1, 4-7) | HTN and DM prioritized and given more attention than HIV | Diabetes can be stressful and can lead to many problems that can make someone sick and bedridden especially thinking of how to get the medications. The many years that I have lived with HIV since 2002 up to now, I have known that HIV cannot kill a person. […] But diabetes will kill you due to stress. | Bosire et al., 2018, pg11 |
|  |  |  |  | “I think it’s better to have HIV than pressure. . .You know with HIV you can just take medication, and you will be fine. You can just continue with your life. But with pressure it can just go up suddenly and you can die instantly.” (Female, 44 years) | Manavalan et al., 2020, pg7 |
|  |  |  |  | Some participants expressed that hypertension for them was more serious than HIV therefore requiring more care: ‘But I saw that by looking, HIV is better than pressure that I have because with pressure, it does go down or anything, I try to eat things I am told to eat but it is always high.’ | Tokwe and Naidoo, 2020, pg6 |
|  | Greater confidence in ART than in NCD medications | **(1, 4, 8)** | (higher adherence to ART than HTN or DM medication)  ART perceived as very efficacious | “I’ve never thought about stopping using my HIV medications because I know I have to use it for the rest of my life. . .But for high blood pressure? I guess maybe there will be a cure or an alternative.” (Female, 55 years) | Manavalan et al., 2020, pg7 |
|  |  |  |  | These HIV drugs have no alternative. I have witnessed many people who decided to stop taking HIV drugs and it was their end…” Female, 52 years | Ottaru et al., 2024 |
|  |  |  |  | Participants also compared HTN/DM medication to ART; stating that in contrast to the management of HIV with only ART, there are other alternatives to manage HTN/DM including resting, lifestyle modification, and the use of perceived cheaper herbal medication. | Ottaru et al., 2024 |
|  |  |  |  | There was also a perception that hypertensive and/  or diabetes medications are ineffective in controlling  HTN/DM.  …I went to the herbal clinic in search of an alternative, hoping to get treated. Because, all these medications (hypertension and diabetes medication)  are just for pain relief and not curative. They (at  the herbal clinic) claimed to have medications that  could cure diabetes completely…” Male, 60 years | Ottaru et al., 2024 |
|  |  |  |  | Some participants discussed their personal experiences recovering from HIV due to ART, which instilled faith in the efficacy of ART. There were also beliefs that despite taking antihypertensive medications, one might still suffer a stroke, whereas taking ART correctly is completely protective against HIV complications: “There is a way you can control hypertension. Not eating salty foods. But after you start taking ART, if you skip you begin to experience some problems. You can even die because the body immunity becomes weak. So, I feel that between high blood pressure medication and ART, the ART medicine is better. You cannot skip ART” (Female, 61 years old) | Hing et al., 2019,  pg39 |
|  |  |  |  |  |  |
| **2. Divergent treatment approaches for HIV and NCD comorbidities amidst managing polypharmacy** | Consistent ART adherence versus intentional or selective non-adherence to NCD medication | (1-4, 9, 10) | Emphasizing consistency and adherence to ART. | "Since I started taking [ART], I see I live a better life. That is why I try my best to take the medicine and why try to remember every day not to forget"(Male, 58 years old) | Hing et al., 2019,  pg40 |
|  |  |  |  | PLHIV choose HIV treatment but refuse treatment for other chronic diseases. | Koffi et al., 2025, pg3. |
|  |  |  | ART perceived as very efficacious | “I’ve never thought about stopping using my HIV medications because I know I have to use it for the rest of my life...” (Female, 55 years) | Manavalan et al., 2020, pg7 |
|  |  |  |  | … But after you start taking ART, if you skip you begin to experience some problems. You can even die because the body immunity becomes weak. So, I feel that between high blood pressure medication and ART, the ART medicine is better. You cannot skip ART (Female, 61 years old). | Hing et al., 2019,  pg39 |
|  |  | (1, 5) | Taking HTN or DM medicines | "I have seen it back then from my parents ... my father died of hypertension, and the younger brother of my father who stays in South Africa had a stroke because of [hypertension], so I don’t for a day miss the [hypertension] drugs" (Male, 52 years old). | Hing et al., 2019,  pg40 |
|  |  |  |  | “You cannot survive without medication … if we have to take a lot of medication, …, so you can’t avoid medication when there is pressure.” 48-year-old female, HTN | Temu et al., 2017, pg5 |
|  |  |  |  | Taking medication constituted a major task for patient-participants for them to maintain optimum health outcomes | Matima, Murphy et al., 2018. Pg9 |
|  |  | (2, 4, 5, 7, 8, 10) | Non- adherence to DM or HTN medicines | "At first, I used to take them every day… Then I saw that I was not having problems with my BP as before, so I stopped. Then I got measured and saw that it had spiked a bit, so I started taking them again". (male, 62y, 4 years on antihypertensive medication, misses ≥1 dose per week) | Moucheraud et al., 2022, pg6-7 |
|  |  |  |  | Observations at home and the long hours I spent with many patients revealed how they poorly adhered to their medication. | Bosire, 2021, pg7 |
|  |  |  |  | In general, participants appeared reluctant towards antihypertensive medications. Participants who reported previous antihypertensive use typically only used their medication when feeling symptomatic or until their prescription ran out. | Manavalan et al., 2020, pg8 |
|  |  |  | Choose not to take NCD medication:  *Choose not to take HTN and DM medication: Due to 1) Lack of medicines; (not getting medicines from facility or unable to buy); 2)*  *side effects of the meds; 3), due to is not experiencing the signs and symptoms of hypertension; and* | Worse still, she is more stressed because of her sick sisters, inability to pay rent, and lack of food—she cannot afford even the cheapest foods; “This is why I have to skip taking my insulin because my body gets weak, I shake and feel like going mad,” | Bosire, 2021, pg6 |
|  |  |  |  | “I’m not using (blood pressure medication) . . . It’s hard to raise the money now. You need money for food. You need money for this medication. If you suddenly get a fever, you need money again, so how are you going to manage it?” (Female, 78 years) | Manavalan et al., 2020, pg9 |
|  |  |  |  | “It is very tough as a bus driver whenever I take the pressure pill I have to go to the toilet all the time so …. I chose to only take it when I feel sick.” (Male)  **“**“I have been prescribed two medications a day, but I usually take one a day because I can’t afford and sometimes none unless I have a headache.” (58 years old) | Temu et al., 2017, pg5 |
|  |  |  |  | They would stop taking the medication immediately after the symptoms disappeared and resume treatment when the symptoms reappeared due to the inability to afford the costs of the medication. One participant said, “…I only seek treatment when I’m ill or if my blood sugar is high. When it gets high, it is when I start buying medication for a month, which I will use until I feel better. If I go and test and see that it has gone back to 11, I stop taking the medication. Sometimes, a month and a half can go by without getting seriously high, then it spikes up again. That is how I live…” Male, 60 years, | Ottaru et al., 2024 |
|  |  |  |  | All patient-participants struggled at the time of diagnosis with the pill burden, but this diminished over time and they eventually did not feel overburdened to manage their health “I found it difficult at the time that I'd just been diagnosed with diabetes. . .to inject myself and I have to take a certain amount of pills. . . but now I became to use to it and now I'm just living life” | Matima, et al., 2018. Pg9 |
|  |  |  |  | “Pressure has caused my sugars [diabetes] to go high. I do inject myself. I, however, do not use insulin so much because, it affects my legs. They get [so] swollen that I can’t walk. So, I just use [it] some days and skips other days.” | Bosire et al., 2018, pg11 |
|  |  |  | Told by religious / church leaders | "Some churches tell their members to stop taking medication, and that they have been prayed for and healed". (female, 67y, 2 years on antihypertensive medication, never misses) | Moucheraud et al., 2022, pg6 |
|  |  |  | *4) told by healthcare worker* | "After they tested [my blood pressure], they told me I am alright and that I need to stop taking the medication". (female, 52y, 1 year on antihypertensive medication, misses ≥1 dose per week) | Moucheraud et al., 2022, pg7 |
|  |  |  |  | "[The doctor] said my condition is stable and I should stop taking the drugs… They will let me know if they see that I need to get back on the drugs, if my BP is high… I do not stop taking the medication for good, but they need to observe me. I am now nearing a year since I stopped taking the medication and my BP level is the same as it was when I was taking the medication". (male, 43y, 4 years on antihypertensive medication, never misses) | Moucheraud et al., 2022, pg7 |
|  | Use of alternative and complementary treatments | (2, 4, 8, 10, 11) | Drinking lots of water | A few participants had tried herbal, natural and traditional treatment including drinking lots of water. | Manavalan et al., 2020, pg8 |
|  |  |  | using herbal plants including eating ginger or garlic supplements, cucumbers, moringa, cinnamon, hibiscus, sweet potato leaves, lemongrass, and mushrooms | I take the mushroom when I see that my heart rate is high. It is from South Africa, and it helps with things like BP, ulcers, body pains. My child got it for me… It’s what I have been using since 2011. You have to buy the mushroom, and it is a bit hard for me [to afford it] … But when I don’t have the mushroom, I know that I am putting my own life at risk. (female, 60y, 2 years on antihypertensive medication, misses ≥1 dose per week) | Moucheraud et al., 2022, pg7-8 |
|  |  |  |  | during our observation of the clinical procedures,  we listened to several patients who shared  ... amongst themselves about drinking and eating bitter  herbs and green vegetables, respectively, in order to manage diabetes and hypertension. During such informal  conversations, patients gave each other advice and were  seen smiling and laughing together. | Akugizibwe et al., 2023. pg7 |
|  |  |  | Use of sour herbs including bitter leaf or 'vernonia amygdalina' and 'momordica foetida' | “I started to take sour herbs like ‘mululuza. (bitter leaf or vernonia amygdalina)’ and ‘bombo’ (momordica foetida). They told me that if I take them the blood pressure will reduce.” (Widowed WLHIV with hypertension, 44 years) | Van Hout et al., 2023, pg7 |
|  |  |  |  | “Because some people say that diabetes will heal when you take herbs, some say it can only be soothed and some herbs soothe.” (Widowed WLHIV with hypertension and diabetes, 50 years) | Van Hout et al., 2023, pg7 |
|  |  |  | Spiritual interventions | "two participants believed that hypertension could be cured through prayer." | Moucheraud et al., 2022, pg6 |
|  |  |  |  | "Some churches tell their members to stop taking medication, and that they have been prayed for and healed". (female, 67y, 2 years on antihypertensive medication, never misses) | Moucheraud et al., 2022, pg6 |
|  |  |  | visiting a traditional healer | A few participants had tried herbal, natural and traditional treatment including drinking lots of water, eating cucumbers, using herbal plants, eating ginger and garlic supplements, and visiting a traditional healer. | Moucheraud et al., 2022, pg8 |
|  |  |  | A combination of alternatives | “…I could use alternative therapies. For example, sometimes when I feel hypertensive, I turn on the TV or Radio and watch or listen to music and I feel good. Or you can chew your garlic, drink plenty of water and sleep. When you wake up in the morning you feel renewed. | Ottaru et al., 2024 |
|  | Stress reduction techniques to manage HTN | (5, 8) | Avoiding stressing relationships; change in social relationships due to HTN | Stress reduction and lifestyle changes were the most frequently cited treatment strategies. A 29-year-old male stated, “Dad does not prefer stress …. That is why we are staying with him … my mom died and I live with my uncle and two children … we are the only people around him and ehh the other drunkard uncles are away in Nairobi … he requests us to talk to them since if his pressure will go up he will die … what my dad tells us is that he doesn’t want to be annoyed because if he does … the heart might stop functioning and he will die.” | Temu et al., 2017, pg5 |
|  |  | (2, 4) | Avoiding stressful environments or experiences such as noise, worries; | "Socially, well I should not think a lot, I should not be at a noisy place, when someone wants to tell me something they should not tell me in a loud frightening way". (female, 49y, 2 years on antihypertensive medication) | Moucheraud et al., 2022, pg8-9 |
|  | Patients’ management of multiple medications | |  |  |  |
|  | i. Strategies to support adherence to the different prescribed medicines | (1-4) | •Taking both types of medication at the same time | There were several practical strategies that participants recognized as facilitating adherence to both hypertension medications and ART. These included taking both types of medications at the same time, | Hing et al., 2019, pg40 |
|  |  |  |  | patient-participants had integrated taking medication into their daily routine included setting alarms to remind them to take their medication and taking medication just before meals. | Matima, et al., 2018. Pg10 |
|  |  |  | •involving family members to provide daily reminders | "I actually tell the boys and guardians that I stay with that they should remind me. When they realize that I have forgotten, they say ‘Mother, you have forgotten your medications’. And you would see that they are bringing me a cup of water so that I take the pills" (Female, 58 years old). | Hing et al., 2019, pg40-41 |
|  |  |  |  | My wife reminds me to take the drugs. (male, 64y, 12 years on antihypertensive medication, misses ≥1 dose per week) | Moucheraud et al., 2022, pg6 |
|  |  |  | •Having a shared "drug bag" | There were several practical strategies that participants recognized as facilitating adherence to both hypertension medications and ART. These included … and having a shared ‘drug bag’ so that all the medications were in the same place in the home. | Hing et al., 2019, pg40 |
|  | ii. Strategies to deal with pill burden: | (4, 5) | Do physical exercises instead of taking HTN pills. | A 58-year-old hypertensive male said, “My doctor asked me if I have been taking pressure drugs and I say no… so he then advises me to go buy and use the drugs he had prescribed until the next visit …. When I went home, I figured …. I am taking too much drugs. The ones for HIV and pressure and I decide to try exercise instead.” | Temu et al., 2017, pg5 |
|  |  |  | Use herbs instead of medicines | “I thought it would be too much for me. I already have this medication (for HIV), and now starting this medication (for hypertension) that I have to take for a long time? I didn’t want to get used to it (antihypertensive medication), and I decided I would stick with more natural methods, and sometimes I even felt better. . . After eating garlic sometimes I would go and check my blood pressure, and I found that my pressure was a bit lower.” (Female, 55years) | Moucheraud et al., 2022, pg8 |
|  |  |  | Omit/ refuse/ opt to stop taking HTN medication. | A 42-year-old female hypertensive summed it all, “Pressure has signs …. I normally know when my pressure is high cause I get migraine on the forehead or I see black … when you see black then you know pressure is abnormal … personally …. I refused medication because there is no way I can take the medication for the virus and then add for pressure … that is too much drugs in your body. I told the doctor …. I will control my pressure because there is a way you can feel it is high.” | Temu et al., 2017, pg4 |
|  |  |  |  |  |  |
| **3. Patients adopted health-promoting behaviors** | Dietary modifications and challenges | (4, 6, 12) | a. Selective of when and what to eat:  i. Eat frequently, Eating idea / small portions of food | Patient-participants also mentioned the importance of ideal portion size and frequency of meals on a given day: “I must eat five times a day. Not like three times a day and making six slices. I must make like small portions. But I must make sure my starch is less and less, because according to her that is causing my weight to rise.” P8, male. | Matima et al., 2018,  pg10 |
|  |  |  | ii. Avoiding certain foods (fatty, salty, and sugary foods) | ‘When you have these conditions, …, you need to be a person who loves drinking water, the food must not have excess of fat, and alot of salt.’ (T3, L 423–435) | Tokwe and Naidoo, 2020, pg6 |
|  |  |  |  | Some respondents spoke about the recommended dietary changes to manage hypertension: avoiding certain foods (red meat, fatty foods, salty foods, and sugars) and eating more vegetables and fruits. | Moucheraud et al., 2022, pg8 |
|  |  |  | b. Drinking a lot of water | A 48-year-old female with a 5-year history of hypertension stated, “You cannot survive without medication … if we have to take a lot of medication, we take a lot of water so that we are able to flush those drugs from the blood stream, so you can’t avoid medication when there is pressure.” | Temu et al., 2017, pg5 |
|  |  |  | c. Eating food before taking medication | First, I take my bath, brush my teeth, and prepare breakfast for my wife and my son. My wife and I will then eat our breakfast at around 8.00 a.m. and take our medication. | Bosire, 2021, pg7 |
|  |  |  |  | Worse still, she is more stressed because of her sick sisters, inability to pay rent, and lack of food—she cannot afford even the cheapest foods; “This is why I have to skip taking my insulin because my body gets weak, I shake and feel like going mad,” | Bosire, 2021, pg |
|  |  |  |  | Several patients admitted being hungry regularly, skipping meals or having to take ART on an empty stomach, which is not recommended. People with diabetes, who were urged to eat fresh, healthy, and non-fatty foods, struggled to adhere to the recommended diet, as fresh fruit and vegetables were unaffordable or would not be available all year around. | Van Pinxteren et al., 2023, pg6 |
|  |  |  |  | ‘She’d encourage me to cook on time so that I can eat your pills (Referring to her mother).’ (T2, L 380) | Tokwe and Naidoo, 2020, pg4 |
|  |  |  | Effect of social norms on food consumption | In fact, going on a healthy diet was a source of ridicule or trivialisation. They had to make great efforts, even sacrifices, in order to adhere to the diet. Social norms about food correspond to a consumption standard of the majority, for example with regard to types of food or portion sizes, and have an influence on their consumption. To adhere to the diet, participants no longer have to conform to their own eating habits and the eating norms accepted by those around them. | Koffi et al., 2025, pg5. |
|  |  |  | d. Resort to eating any type of food | However, despite the goodwill of others, some families struggled to put food on the table, which impacted their health. | Van Pinxteren et al., 2023, pg6 |
|  |  |  |  | Some patients were unable to get rid of certain food cravings that are prohibited in the healthy diet. As a result, some of the diet’s restrictions were respected, while others were not. | Koffi et al., 2025, pg5. |
|  |  |  |  | “I need more nutritious food, but we cannot always afford this kind of food. It is tough as I told you. So, the doctors think I am not committed to improving my health but what do I do if there is no money? In fact, this whole thing about eating healthy begins to irritate me because they act like they cannot see this rural area, there is nothing. This food they speak of, where are we supposed to get it?” PR014 [M34] | Van Pinxteren et al., 2023, pg6-7 |
|  |  |  |  | For some respondents, particularly widowed women, the recommended dietary changes were seen as restrictive and difficult to adhere to. Respondents said that healthy foods are difficult to find, afford, or prepare. "When there is no one around, I go to sleep without eating because there is no one to buy food for me". (female, 70y, 4 years on antihypertensive medication, misses ≥1 dose per week) | Moucheraud et al., 2022, pg8 |
|  |  |  |  | “ …your rice is not right, potatoes are not right, mealies are not right and that is the only food that we eat. So, we can't afford that stuff.” | Matima, Murphy et al., 2018, pg8 |
|  |  |  |  | Two male respondents spoke about how they have made changes in their diet at the household level and feel as though this is difficult for others in the family who do not need to eat a restricted diet. "What has changed really its diet. My wife at home doesn’t allow that they should have their own food and I should also eat mine, so we just eat what I eat and I feel like it’s not fair to her". (male, 64y, 12 years on antihypertensive medication, misses ≥1 dose per week) | Moucheraud et al., 2022, pg8 |
|  |  |  |  | … adherence to recommendations was often challenging. Except for one patient-participant, all participants reported that they shared the same food with their families and struggled to afford the recommended foods. In most cases, this situation forced patient-participants to forego the ideal diet for their chronic conditions: “ . . .your rice is not right, potatoes are not right, mealies are not right and that is the only food that we eat. So we can't afford that stuff.” Participant P2, female | Matima et al., 2018,  pg8 |
|  |  |  |  | “. . .sometimes she [wife] has to make two pots, because when I say to her, ‘Please when you cook for me, cut the skin before you cook’ she is not diabetic [and] she says, ‘The meat is much more enjoyable with the skin.’ So it’s a challenge in every family because being diabetic you adjust your life, but when you are not it’s not easy.” Participant P8- male | Matima et al., 2018, pg8 |
|  |  |  |  | “I’m not using (blood pressure medication) ... It’s hard to raise the money now. You need money for food. You need money for this medication. If you suddenly get a fever, you need money again, so how are you going to manage it?” (Female, 78 years) | Moucheraud et al., 2022, |
|  | Lifestyle adjustments | (3, 6, 9, 10, 12) | Cease smoking  and alcohol use | ‘When you have these conditions, you don’t drink alcohol, you don’t smoke cigarette,...’ (T3, L 423–435) | Tokwe and Naidoo, 2020, pg6 |
|  |  |  |  | ‘I do drink alcohol but I don’t smoke....’ (T7, L 516–518) | Tokwe and Naidoo, 2020, pg6 |
|  |  |  | Physical exercise/ Exercising for weight loss to manage DM | Three patient-participants mentioned that they had developed a daily routine of walking in order to manage T2D. Two other patient-participants said that they danced or jogged to lose weight: “There was a time that my weight used to be hundred and ten [kilograms], but now, ever since I started exercising it's gone down to ninety-four, so exercising helps. Even if you don't have to walk around the place—just being at home and dancing is exercise.” - P3, female | Matima et al., 2018, pg10 |
|  |  |  |  | Physical activity is seen by participants as a factor contributing to physical well-being. | Koffi et al., 2025,pg4 |
|  |  |  |  | Some participants, especially younger participants focused on lifestyle modification such as engaging in physical exercise and adopting a balanced diet. | Ottaru et al., 2024 |
|  |  |  | Taking immune boosters | “I am paying for the boosters, vitamins, and stuff. To boost my body. At the pharmacy, they sell it for more than 30 rand, so I buy one at the time.” PU007 [F48] | Van Pinxteren et al., 2023, pg6 |
|  | Condition-specific health promoting practices | (4, 12) | Protecting open wounds against infection | Patient-participants practiced personal safety for both HIV and T2D. For HIV, patient-participants spoke about safer sexual practices and the need to protect open wounds. | Matima et al., 2018, pg10 |
|  |  |  | Foot care for DM: washing and drying feet properly; wearing the right shoes | for T2D, one patient-participant mentioned the need for proper foot care, which included washing and drying feet properly; and also wearing the right shoes "to be safe" | Matima et al., 2018, pg10 |
|  |  |  | Safer sexual practices for HIV | For HIV, patient-participants spoke about safer sexual practices and the need to protect open wounds . | Matima et al., 2018, |
|  |  |  | Blood pressure and glucose levels monitoring. | “I check my blood pressure once in a while because sometimes I may receive some scary news and hear things that worry me. And when things like that happen my heart starts beating fast and I get short of breath. When I feel like that I go to the dispensary and check my pressure.” (Female, 45 years). | Manavalan et al., 2020, pg7 |
|  |  |  |  |  |  |
| **4. Coping with psychosocial distress** | Psychosocial coping strategies. | | |  |  |
|  | Acceptance as a copying mechanism | (3, 6, 12, 13) | Developing a positive attitude towards chronic illnesses | “That time that I just found out this condition, that I’ve got these diseases. . .I felt very, very low at that time. I felt like I couldn't accept the way that I had to live, but as time went by, I accepted those things and that's what I am now.” Participant P1, female. | Matima et al., 2018, pg11 |
|  |  |  |  | I feel nothing about it because I was counselled and told that I am not the only with the condition. We are many having it (47-yr- old Female, DM&HTN). ...  Mmm in our daily life there are some people who call it “mpawo atalikaaba” which means that every person has cried because of HIV, it has reached every family. But me I’m okay with it. I don’t care what they say because I know that I’m not a thief but a patient looking for life support. I talk freely because now I’m used to the people since they already know that I have all the diseases (50-yr-old Female, HIV, DM &HTN). | Akugizibwe et al., 2023 pg7 |
|  |  |  |  | Participants identified that overcoming the various forms of stigma and discrimination was a process that varied for each participant and was dependent on their acceptance of their HIV or hypertension diagnosis: ‘I was scared that they will gossip and say things you see. I don’t know this condition; it is not lucky.’ (T1, L 641–642) | Tokwe and Naidoo, 2020, pg3 |
|  |  |  |  | “The best I can do is to adhere to the guidance from the nurses and not miss my clinic dates and take my medication duly. I have no stress about my conditions because I have accepted them.” PU008 [M56] | Van Pinxteren et al., 2023, pg4 |
|  | Managing stigma | (2, 6, 12) | Hiding medicines | In addition, some participants towards a new normal of living with HIV experienced that when they were going in places where there were people, they would remove their medications from their containers to minimise the noise caused by the pills that attracts attention of people in their community and results in stigmatization: ‘Sometimes I put it inside the container and wrap it with a toilet paper so that it stops the movement that makes the noise.’ (T9, L 734–735) | Tokwe and Naidoo, 2020, pg4 |
|  |  |  | Not disclosing the disease | “I didn’t tell anyone. I don’t want to tell them, because sometimes even my family is not alright. If you clash, they say this guy has it [HIV]. . .but they know I’ve got diabetes and the way they talk hurts. [They say] ªWhere are you getting the diabetes when you are small?” I tell them I did not call for the sickness.” | Matima et al., 2018, pg9 |
|  |  |  | Staying away from home | “My brothers stigmatize me a lot. Sometimes when I want to go to bed, they insult me the whole night. Sometimes they take stones and throw them on the roof, and I feel like I am shocked and fall down. So sometimes I don’t even stay at my own home.” (Female, 44years) | Manavalan et al., 2020, pg9 |
|  | Maladaptive coping | (5, 6, 8) | drinking alcohol | The participants verbalised the experience of change in the lifestyle of adopting unhealthy addictions as a way to coping with living with HIV and hypertension. The change was verbalised as developing an addiction to smoking and heavy alcohol intake that they experienced as a barrier towards the lifestyle: ‘I do drink alcohol, but I don’t smoke. No, I do drink alcohol. Yes I sometimes have that problem when I drink alcohol day before going to clinic you know and my blood pressure becomes high you know. So this is where I suspect that I am using alcohol heavily sometimes I’ve seen that my pressure becomes high when I have used alcohol heavily.’ (T7, L 516–518). | Tokwe and Naidoo, 2020, pg6 |
|  |  |  |  | *some participants resorted to maladaptive coping strategies.* | Ottaru et al., 2024, pg |
|  | *Before presenting theme six, we provide a preamble to the health insecurities faced by patients* | | | |  |
|  | *Preamble to healthcare insecurities that were observed across the studies* | 1. *Disparity in resource availability* | *Access to diabetes and hypertension drugs was illustrated as a recurrent problem for all because, while HIV drugs was freely available at no cost, these non-communicable disease medications were at the personal cost of the patient. ... One described inconsistent pricing of diabetes and hypertension drugs (ranging from 1000Ush to 1500Ush per tablet (approximately 0.28 – 0.43 USD). This was said to hinder adherence to treatment regimes. “…a challenge is that the medicine is expensive… here at the clinic they don’t have the medicine for blood pressure, so sometimes I’m able to buy and sometimes I don’t afford it.” (Widowed WLHIV with hypertension, 44 years)* | | Van Hout et al., 2023, pg9 |
|  |  |  | *Lack of proper care for people with diabetes influenced the way they experienced their illnesses as well as how they perceived those suffering from HIV and AIDS.* | | Bosire et al., 2018, pg11 |
|  |  |  | *There is no diabetes or nutrition department at the HIV clinic, however, the centre does have a diet sheet listing the foods that should and should not be consumed by patients with a high body mass index (BMI) or high blood sugar.* | | Koffi et al., 2025,pg6 |
|  |  |  | *...people living with HIV (PLWHIV) with very low body-mass index (BMI) were referred to the dietician for a food programme for dietary supplementation. Additionally, external advocacy groups stationed permanently at HIV clinics such as Treatment Action Campaign (TAC) championed the rights for all PLWHIV and Mothers2Mothers had empowering programmes for mothers living with HIV.* | | Matima et al., 2018,  pg12 |
|  |  | 2. *Uneven access to quality NCD care and health information compared to HIV* | *Participants felt that HIV information was more easily available, as they received counselling after their HIV diagnosis. However, health education and counselling after diagnosis of other chronic conditions, was scarce. “I receive a lot of pamphlets and information about HIV, but I do not have much about my other condition ... I would like to know more about how to manage my other conditions.” PU002 [F62]* | | Van Pinxteren et al., 2023, pg8-9 |
|  |  |  | *“We have free ARV medication and good doctors. The clinic [HIV clinic] offers good medical care and counseling to the patients.” She also adds that “HIV is ok because once someone starts medication, everything goes on well.” The counseling and care she receives at the HIV clinic has helped her manage her HIV well, compared to her other diseases. She adds: “HIV is better than diabetes and pressure because, HIV is easier to manage but the others are difficult to manage.” This difficulty is not due to the symptoms of diabetes and hypertension but rather insufficient clinical support and care for these diseases.* | | Bosire et al., 2018, pg10 |
|  |  |  | *High transportation costs Participants reported incurring a significant financial burden while accessing care for HTN/DM in addition to care for HIV infection at different health facilities.* | | Ottaru et al., 2024, pg10 |
|  |  |  | *For instance, Grace can access comprehensive care for her HIV, but her diabetes and high blood pressure are neglected within the health care system and neglected within her own self-care priorities. Grace’s case is not a unique one as others described the same concern, as exemplified by a 55-year-old man with diabetes: If you look at the government, it hasn’t provided free drugs for diabetes. You have to buy [them]. If you go to the pharmacy here at the hospital, they will tell you there are no drugs go to the chemist, and you know our drugs [diabetic medicines] are very expensive.* | | Bosire et al., 2018, pg11 |
|  |  |  | *Respondents reported receiving minimal counseling from their healthcare providers regarding the causes, symptoms, complications and treatment of hypertension. When medications were prescribed, respondents noted they often did not receive counseling regarding side effects, potential drug-drug interactions, treatment duration or medication adherence. Participants reported that plans for blood pressure monitoring and follow-up were often not discussed.* | | Manavalan et al., 2020, pg8 |
|  |  | 3. *Disparity in healthcare funding /support* | *“We have free ARV medication and good doctors.....” She also adds that “HIV is ok because once someone starts medication, everything goes on well.”* | | Bosire et al., 2018, pg10 |
|  |  |  | *... She adds: “HIV is better than diabetes and pressure because, HIV is easier to manage but the others are difficult to manage.” This difficulty is not due to the symptoms of diabetes and hypertension but rather insufficient clinical support and care for these diseases.* | | Bosire et al., 2018, pg10 |
|  |  |  | *Existence of supported HIV care programs and clubs accelerated HIV selfcare* | *“....The clinic [HIV clinic] offers good medical care and counseling to the patients.” The counseling and care she receives at the HIV clinic has helped her manage her HIV well, compared to her other diseases.* | Bosire et al., 2018, pg10 |
|  | *Below is matrix of theme six showing how patients attempted to navigate the insecurities*. | | |  |  |
| **5.Strategies to navigate healthcare insecurities** | Navigating limited availability of NCD treatments | (1, 3, 4, 7, 9-12) | Patients buying their own supplies out of pocket | Five urban participants complained that it was difficult to rely on the health services because of frequent stock outs of medication, which interrupted their self-management routines. The unavailability of monitoring tools was also reported, including blood pressure monitors and glucometers, and pillboxes to organize medication, which compromised their ability to control their conditions. Participants had to either buy their own supplies out of pocket or had to travel to the clinic to measure their blood pressure or glucose levels, which is time consuming and expensive. | Van Pinxteren et al., 2023, pg8 |
|  |  |  |  | Thus, one may perceive non-communicable disease care to be systematically excluded from the health system as out-of-pocket payments can fundamentally deter health seeking | Bosire et al., 2018, pg11 |
|  |  |  |  | HIV donor funding has contributed to vertically controlled HIV programs, which are siloed into targeted clinics, medication, and care that is not fully integrated within the health system at the district hospital where we worked. This has in some sense contributed to fragmentation of chronic care for other non-communicable diseases such as diabetes because these non-priority diseases require out-of-pocket payments. | Bosire et al., 2018, pg11 |
|  |  |  | Patients visited / attended different health facilities in search of medicines. | Although diabetes and hypertension were managed together in one clinic, patients had to visit other clinics for any other illness that they had, including diabetes complications ... some patients attended both PHC clinics and tertiary hospital, although comprehensive HIV care was provided at PHC clinics, comprehensive diabetes care was only provided at the tertiary hospital. Some of the reasons provided for not accessing comprehensive diabetes care at the PHC clinics included drug stock-out, lack of doctors, and unskilled nurses. | Bosire, 2021, |
|  |  |  |  | Participants reported utilizing lower-level health facilities and community pharmacies near the homes to monitor their blood pressure and sugar levels instead of making visits to the health facilities where they are required to pay for doctor’s consultations and transport fee. A | Ottaru et al., 2024, pg10 |
|  |  |  | Patients substituting unavailable medications with pain killers  Borrowing  medication for DM | Sometimes, pain tablets would not last the whole month and had to be substituted with aspirin or borrowed medication from others: “Right now, I am out of pills. I have asked my friend for pain pills. Mine ran out last week, I will only get more in April.” PU002 [F62]. | Van Pinxteren et al., 2023, pg6 |
|  |  |  | Endure living without taking medicines and disease monitoring | When discussing the financial dimensions of hypertension adherence, participants contrasted them unfavourably to the ease with which they access HIV care, particularly free ART. Individuals expressed worry and anxiety in knowing that they had a health issue—hypertension—but were unable able to take action on it because of money. "Taking [hypertension] medicine is not a choice but a problem where I cannot find the medicine, while the ART, I have never been in a situation that I want to go get the drugs but find that there is no medicine at the hospital . . . all times they are there and are free" (Male, 51 years old). | Hing et al., 2019, pg 39 |
|  |  |  |  | Some observed having to live with their symptoms until they had funds to purchase the medicines. “A challenge is lack of drugs. …There are times when they do not have drugs for diabetes. They give you one pack and tell you the buy the rest. You may not have money for buying drugs at that particular time.” (Married WLHIV with diabetes, 42 years) | Van Hout et al., 2023, pg9-10 |
|  |  |  |  | Participants reported that HIV and hypertension care were managed separately. Participants revealed that their HIV providers did not know they were hypertensive and that their hypertension providers did not know they had HIV. The majority noted that their blood pressure was never measured in the HIV clinic. …“No, I’ve never checked my blood pressure at the HIV clinic...” (Female, 39 years) | Manavalan et al., 2020, pg9 |
|  | Seeking multiple sources of care and information | (3, 4, 7, 8, 10, 12) | Actively search for healthcare information | “It also depends on your curiousness. If you are not curious enough then the information is going to be far away from you. You have to be inquisitive ….” P8, male. | Matima et al., 2018, pg11 |
|  |  |  | ***Attending multiple clinics for:*** | |  |
|  |  |  | • Consultation and treatment of each condition | Observations in this clinic revealed how patients with multimorbidities moved from one clinic to another for consultations and treatment, a process that was said to be overwhelming and confusing. | Bosire, 2021, pg4 |
|  |  |  |  | “I visit two separate clinics for my diseases: HIV clinic and a diabetes clinic for diabetes and hypertension.” Participant Y | Bosire, 2021, pg7 |
|  |  |  |  | “These days, my CD4 count has improved and I collect my ARVs from a local clinic in my neighborhood. However, I still attend Bara for my diabetes care because, my diabetes is uncontrolled.” | Bosire, 2021, pg6 |
|  |  |  | •Diabetes complications | Although diabetes and hypertension were managed together in one clinic, patients had to visit other clinics for any other illness that they had, including diabetes complications (e.g., podiatry and eye clinics). | Bosire, 2021, pg4 |
|  |  |  | • Information | …, visiting numerous uncoordinated clinics led to patients receiving conflicting information from clinicians. Participants revealed that clinicians rarely collaborated or communicated while managing their multiple illnesses … “The problem is that one doctor will tell you to do this, and another asks you to do a different thing.” (A 64-year-old woman (HIV, HTN, DM, diabetes)) | Bosire, 2021, pg4 |
|  | Financial coping strategies to sustain self-care | (3, 7, 10-12) | Extra work / seeking additional income | A few patients took up extra work or rented out rooms to raise money to buy the recommended food. “I am getting an old age grant now. But I cannot say I am okay (financially), so I am selling sheep heads to add more, because I want to eat veggies like broccoli and spinach, the fresh foods.” PU005 [F63] | Van Pinxteren et al., 2023, pg6 |
|  |  |  |  | “I always try to ensure that I collect wood to sell so I can go to the clinic…. I need R100 to go and come back, even if I do not eat, it’s fine.” PR06 [F46] | Van Pinxteren et al., 2023, pg7 |
|  |  |  | Borrowing money to meet medical bill | “I always try to ensure that I collect wood to sell so I can go to the clinic. Or if I cannot find anyone to sell to, I borrow money, even if I do not know how I am going to return it. I need R100 to go and come back, even if I do not eat, it’s fine.” PR06 [F46] | Van Pinxteren et al., 2023, pg7 |
|  |  |  | Seeking money from social circles | One tablet costs 1000-1500TZS ($0.45-$0.65). My children and relatives usually help me buy a few medications at a time…that is I only buy for 10,000TZs ($4) and when they are finished, I buy again for 10,000TZS” Female, 46 years. | Ottaru et al., 2024, pg10 |
|  |  |  | Selling of personal items | Some described having to sell something from home, in order to have sufficient money for diabetes and/or hypertension treatment. | Van Hout et al., 2023, pg9 |

**REFERENCES**

1.	Hing M, Hoffman RM, Seleman J, Chibwana F, Kahn D, Moucheraud C. 'Blood pressure can kill you tomorrow, but HIV gives you time': illness perceptions and treatment experiences among Malawian individuals living with HIV and hypertension. . Health Policy Plan. 2019;34(Suppl. 2).

2.	Moucheraud C, Phiri K, Hoffman RM. Health behaviours and beliefs among Malawian adults taking antihypertensive medication and antiretroviral therapy: A qualitative study. Global Public Health. 2022;17(5):688-99.

3.	Van Pinxteren M, Mbokazi N, Murphy K, Mair FS, May C, Levitt N. The impact of persistent precarity on patients' capacity to manage their treatment burden: A comparative qualitative study between urban and rural patients with multimorbidity in South Africa. Frontiers in Medicine. 2023;10(no pagination).

4.	Manavalan P, Minja L, Wanda L, Hertz JT, Thielman NM, Okeke NL, et al. "It's because I think too much": Perspectives and experiences of adults with hypertension engaged in HIV care in northern Tanzania. PLoS One. 2020;15(12 December).

5.	Temu TM, Bahiru E, Bukachi F, Bloomfield GS, Muiruri P, Farquhar C. Lay beliefs about hypertension among HIV-infected adults in Kenya. Open Heart. 2017;4(1).

6.	Tokwe L, Naidoo JR. Lived experiences of human immunodeficiency virus and hypertension in the Eastern Cape, South Africa. African Journal of Primary Health Care & Family Medicine. 2020;12(1).

7.	Bosire E, Mendenhall E, Omondi GB, Ndetei D. When Diabetes Confronts HIV: Biological Sub-citizenship at a Public Hospital in Nairobi, Kenya. Med Anthropol Q. 2018;32(4):574-92.

8.	Ottaru TA, Wood CV, Butt Z, Hawkins C, Hirschhorn LR, Karoli P, et al. “I only seek treatment when I am ill”: experiences of hypertension and diabetes care among adults living with HIV in urban Tanzania. BMC Health Serv Res. 2024;24(1):186.

9.	Koffi JJ, Plaisy MK, Djaha J, Minga A, N’goran S, Boni SP, et al. Perceptions and management of diabetes and obesity among people living with HIV in Côte d’Ivoire: a qualitative study. BMC Public Health. 2025;25(1):555.

10.	Bosire EN. Patients' Experiences of Comorbid HIV/AIDS and Diabetes Care and Management in Soweto, South Africa. Qual Health Res. 2021;31(2):373-84.

11.	Van Hout MCA, Zalwango F, Akugizibwe M, Namulundu Chaka M, Bigland C, Birungi J, et al. Women living with HIV, diabetes and/or hypertension multi-morbidity in Uganda: a qualitative exploration of experiences accessing an integrated care service. Journal of Integrated Care. 2023;31(1):86-99.

12.	Matima R, Murphy K, Levitt NS, BeLue R, Oni T. A qualitative study on the experiences and perspectives of public sector patients in Cape Town in managing the workload of demands of HIV and type 2 diabetes multimorbidity. PLoS One. 2018;13(3).

13.	Akugizibwe M, Zalwango F, Namulundu CM, Namakoola I, Birungi J, Okebe J, et al. "After all, we are all sick": multi-stakeholder understanding of stigma associated with integrated management of HIV, diabetes and hypertension at selected government clinics in Uganda. BMC Health Serv Res. 2023;23(1):20-.
